# Characterization of Macro- and Microalgae Extracts Bioactive Compounds and Micro- and Macroelements Transition from Algae to Extract

**DOI:** 10.3390/foods10092226

**Published:** 2021-09-19

**Authors:** Ernesta Tolpeznikaite, Vadims Bartkevics, Modestas Ruzauskas, Renata Pilkaityte, Pranas Viskelis, Dalia Urbonaviciene, Paulina Zavistanaviciute, Egle Zokaityte, Romas Ruibys, Elena Bartkiene

**Affiliations:** 1 Faculty of Animal Sciences, Institute of Animal Rearing Technologies, Lithuanian University of Health Sciences, Mickeviciaus Str. 9, LT-44307 Kaunas, Lithuania; ernesta.tolpeznikaite@lsmuni.lt (E.T.); paulina.zavistanaviciute@lsmuni.lt (P.Z.); egle.zokaityte@lsmuni.lt (E.Z.); 2 Institute of Food Safety, Animal Health and Environment “BIOR”, Lejupes iela 3, Zemgales priekšpilsēta, LV-1076 Riga, Latvia; vadims.bartkevics@bior.gov.lv; 3 Department of Anatomy and Physiology, Faculty of Veterinary, Lithuanian University of Health Sciences, Mickeviciaus Str. 9, LT-44307 Kaunas, Lithuania; modestas.ruzauskas@lsmuni.lt; 4 Faculty of Veterinary, Institute of Microbiology and Virology, Lithuanian University of Health Sciences, Mickeviciaus Str. 9, LT-44307 Kaunas, Lithuania; 5 Marine Research Institute, Klaipėda University, Universiteto Ave. 17, LT-92294 Klaipėda, Lithuania; renata.pilkaityte@apc.ku.lt; 6 Lithuanian Research Centre for Agriculture and Forestry, Institute of Horticulture, Kauno Str. 30, LT-54333 Babtai, Lithuania; pranas.viskelis@lammc.lt (P.V.); dalia.urbonaviciene@lammc.lt (D.U.); 7 Department of Food Safety and Quality, Faculty of Veterinary, Lithuanian University of Health Sciences, Mickeviciaus Str. 9, LT-44307 Kaunas, Lithuania; 8 Institute of Agricultural and Food Sciences, Agriculture Academy, Vytautas Magnus University, K. Donelaicio Str. 58, LT-44244 Kaunas, Lithuania; romas.ruibys@vdu.lt

**Keywords:** Baltic Sea macroalgae, microalgae, spirulina, antimicrobial characteristics, antioxidant properties, trace elements

## Abstract

The aim of this study was to evaluate the characteristics of macroalgae (*Cladophora rupestris*, *Furcellaria lumbricalis*, *Ulva intestinalis*) and microalgae (*Arthrospira platensis* (Sp1, Sp2), *Chlorella vulgaris*) extracts, including micro- and macroelement transition to extract, antioxidant, antimicrobial properties, the concentrations of chlorophyll (-a, -b), and the total carotenoid concentration (TCC). In macroalgae, the highest TCC and chlorophyll content were found in *C. rupestris*. In microalgae, the TCC was 10.1-times higher in *C. vulgaris* than in Sp1, Sp2; however, the chlorophyll contents in *C. vulgaris* samples were lower. A moderate negative correlation was found between the chlorophyll-a and TCC contents (r = −0.4644). In macroalgae extract samples, *C. rupestris* and *F. lumbricalis* showed the highest total phenolic compound content (TPCC). DPPH antioxidant activity and TPCC in microalgae was related to the TCC (r = 0.6191, r = 0.6439, respectively). Sp2 extracts inhibited *Staphylococcus haemolyticus*; *C. rupestris*, *F. lumbricalis*, *U. intestinalis*, and Sp2 extracts inhibited *Bacillus subtilis;* and *U. intestinalis* extracts inhibited *Streptococcus mutans* strains. This study showed that extraction is a suitable technology for toxic metal decontamination in algae; however, some of the desirable microelements are reduced during the extraction, and only the final products, could be applied in food, feed, and others.

## 1. Introduction

The increasing demand for natural ingredients for the food, feed, nutraceutical, and other industries has led to broader utilization of micro- and macroalgae as natural sources of valuable bioactive compounds. Nowadays, biotechonomy includes different biomass valorisation processes to prepare value-added products [1,2,3]. Especially, marine macroalgae is a good alternative to replace terrestrial biomass because it does not compete with plants for uses as food and (or) in feed preparation, and it does not require special resources to accumulate biomass [4]. For this reason, different technologies are used to valorise macroalgae biomass, and different varieties of macroalgae are used to prepare extracts, which are being studied with the prospect of their use in the food, feed, pharmaceutical, cosmetic, and other industries [5].

Algae pigments are very important in plant physiology and are classified into three main groups: chlorophylls, carotenoids, and phycobilins [6]. The main role of the carotenoids is to pass light energy to chlorophyll and to act as very strong antioxidants [7]. *Cladophora* species (Chlorophyta) are a good source of chlorophylls (a and b) and carotenoids, including lutein, β-carotene, and zeaxanthin [8]. According to Khuantrairong and Traichaiyaporn [9], in cultivated *Cladophora* sp. biomass, the β-carotene content could vary from 6.0 to 34.0 μg g^−1^, the lutein content from 125.0 to 221 μg g^−1^, and the zeaxanthin content from 12.1 to 37.1 μg g^−1^. Nazarudin et al. [7] reported a chlorophyll b concentration of 292.52 ± 8.84 μg g^−1^ d.w. in *U. intestinalis*. According to Joyce and Phinn, the highest chlorophyll a content is in *U. intestinalis,* and the lowest is in the brown seaweed *Sargassum ilicifolium* [10]. It was established that in wild-collected algae, the highest concentrations of chlorophyll a and carotenoids are in brown and green algae, and these differences belong to different species with different assimilation capacities for ammonium [11]. Additionally, pigmentation of algae can be related to stress conditions; usually, the concentration of carotenoids increases, and that of chlorophylls decreases under environmental stress conditions [12]. Many factors (water movement, nutrients, light, temperature, salinity, etc.) have an influence on pigment production in macroalgae [13]. The Baltic Sea ecosystem is very sensitive, and strong gradients in salinity and temperature are a big challenge for macroalgae species [14]. It was reported that temperature is the most important factor influencing pigment concentration in macroalgae [15]. According to Vahtmäe et al. [15], the carotenoid concentration in *U. intestinalis* (Chlorophyta) is 162.0 μg g^−1^ d.w., and the highest overall carotenoid concentration is present in green (Chlorophyta) and red (Rhodophyta) algae in comparison with brown algae (Ochrophyta, Phaeophyceae) [16]. However, some studies published that the carotenoid content is high in brown and low in green algae [17]. Similarly, the concentration of pigments can vary depending on the morphological structures of the algae and environmental factors [18]. In addition, *Chlorella vulgaris* (Chlorophyta) and *Arthrospira platensis* (formerly *Spirulina platensis*) (Cyanobacteria) are good sources of various pigments [19].

In addition, the main antioxidants in algae are chlorophylls, carotenoids, fucoxanthin, enzymes, vitamins (E and C), mycosporine-like amino acids, and polysaccharides [20,21,22]; however, the most important are polyphenols [8]. The TPC of marine algae depends on environmental conditions, of which the most important are nutrient concentration, salinity, and UV radiation [23,24,25]. Moreover, in cultivated algae, a higher content of TPC could be related to various stress factors [11].

Although macroalgae are known as a good source of functional compounds [8], they are also known as bioaccumulators of pollution [26], and their ability to accumulate heavy metals, pesticides, dioxins, etc. [26,27,28,29,30], is affected by various factors [8]. Our previous studies showed that Baltic Sea macroalgae cannot be used in food, feed, nutraceuticals, etc., without pre-treatment because of its high degree of contamination with pathogenic bacterial strains and heavy metals [31], and fermentation processing with lactic acid bacterial strains possessing antimicrobial properties is not a sufficient method to avoid biocontamination in Baltic Sea macroalgae. For this reason, in this study, we hypothesised that the preparation of extracts of Baltic Sea macroalgae (*Furcellaria lumbricalis*—Rhodophyta, *Cladophora rupestris,* and *Ulva intestinalis*—Chlorophyta) could lead to heavy metal concentration reduction in algal biomass and could be a prospective method for Baltic Sea macroalgae valorisation to a higher antioxidant potential and new ingredients with broader antimicrobial activity. 

It has been reported that separate compounds in macroalgae and their extracts exhibit anticancer, antineoplastic, antioxidant, and antidiabetic activities, among others [32,33,34,35,36,37]. Munir et al. [34] reported on the antibacterial, antifungal, anti-parasitic, anticancer, immunomodulatory, etc., activities of macroalgae compounds.

In addition to macroalgae, microalgae are becoming an important source of bioactive compounds for many industries. Although microalgae have several attractive characteristics for the preparation of sustainable food, feed, nutraceuticals, etc., only a few varieties have generally been recognized as having safe (GRAS) status according to the Food and Drug Administration (FDA): *Arthrospira platensis*—Cyanobacteria, *Chlamydomonas reinhardtii*, *Auxenochlorella protothecoides*, *Chlorella vulgaris*, *Dunaliella salina* (formerly *Dunaliella bardawil*)—Chlorophyta, and *Euglena gracilis*—Euglenozoa [38]. Nowadays, one of the main problems facing the effective application of microalgae on an industrial scale is insufficient technology. For this reason, in microalgae extract evaluation, antimicrobial properties are paramount, as they could be among the characteristics that could determine the feasibility of industrial-scale application.

The aim of this study was to evaluate the characteristics of macroalgae (*C. rupestris*, *F. lumbricalis,* and *U. intestinalis*) and microalgae (*Arthrospira platensis* (Sp1 and Sp2) and *C. vulgaris*) extracts, including micro- and macroelement transition to extract, as well as antioxidant and antimicrobial properties. In addition, the concentrations of chlorophylls a and b as well as the total carotenoid concentration (TCC) in algae were established. 

## 2. Materials and Methods

### 2.1. Macro- and Microalgae Samples Used in Experiments

**Figure 1:**
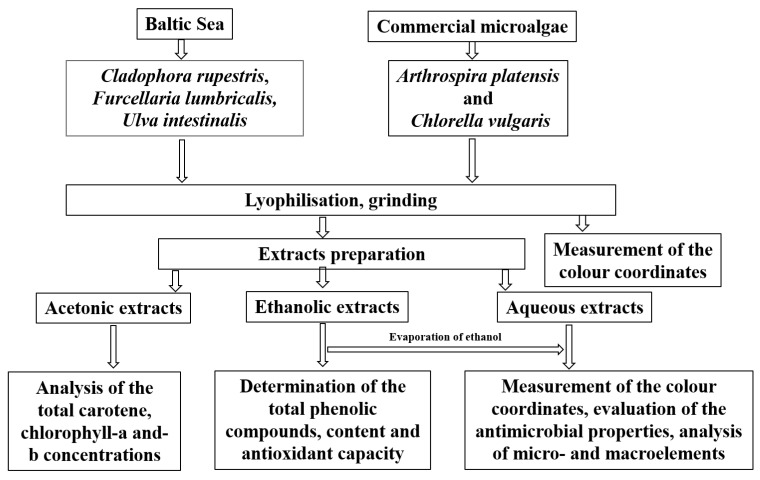
Principal scheme of the experiment.

Principal scheme of the experiment is given in Figure 1.

Samples of macroalgae were collected in the autumn of 2020 on the Lithuanian coast. *U. intestinalis* samples were taken from stones near the surface, while *F. lumbricalis* and *C. rupestris* samples were taken after the storm along the shore. The collected samples were cleaned three times in distilled water to remove sand and macroscopic invertebrates.

In addition, two samples of microalgae (Sp1—Spirulina (*Arthrospira platensis*) from University of Texas Biological Labs (Austin, TX, USA) and Sp2—Spirulina (Ltd. “Spila“, Vilnius, Lithuania, origin from Irvine, CA, USA) were tested. Starter cultures of Sp1 were multiplied according to instructions given by producer. 

All algal samples used in experiments were lyophilized using a freeze-dryer FD8512S (ilShin^®^ Europe, Ede, The Netherlands) and ground into a powder (particle size < 0.2 mm) using a knife mill GM200 (Retsch, Düsseldorf, Germany). Freeze-dried samples were maintained at room temperature in the dark until use.

### 2.2. Chlorophyll a and b and Carotenoid Analysis in Algal Samples

Freeze-dried algal samples of 500 ± 2 mg were removed and weighed accurately. The samples were transferred carefully to a ceramic pestle, and 1.5 mL of ultrapure water was added for sample rehydration. The pestle was covered with aluminium foil for 2 min. The rehydrated sample was ground accurately with a mortar and pestle with 2 g of pure quartz sand. The pigments were extracted and transferred to a volumetric flask, and the volume was adjusted to 100 mL with an 80% solution of aqueous acetone. The homogenised sample mixture was centrifuged at 10,000 rpm for 15 min at 4 °C. The supernatant was separated and immediately analysed.

The acetonic solution mixture was analysed for total carotenoids, chlorophyll a, and b and their derivatives in a spectrophotometer by the modified method of Dere et al. [39], Sakalauskaitė et al. [40], and Sumanta et al. [41]. These compounds were determined at a wavelength of 470 nm after subtracting the concentrations of chlorophyll a and b, using wavelengths of 649 and 665 nm, respectively, and the corresponding absorption coefficients at which carotenoids do not absorb [39,41]. The contents of total carotenoids, chlorophyll a and b, and their derivatives were determined spectrophotometrically, the absorption was measured using a Cintra 202 spectrophotometer (GBC Scientific Equipment Pty Ltd., Mulgrave Victoria Australia), and the results were analysed using the Cintral ver.2.2 program (GBC Scientific Equipment Pty Ltd., Mulgrave Victoria, Australia).

### 2.3. Preparation of Algal Sample Extracts and Determination of the Total Phenolic Compound Content and Antioxidant Capacity of the Algae Extracts

Five grams of the freeze-dried algal sample were extracted with 100 mL of ethanol/water (70:30 *v*/*v*) by incubation at room temperature overnight with stirring. Then, extracts were centrifuged at 3500 rpm for 10 min at 4 °C and filtered through Whatman No. 4 filter paper. Ethanol in the solvent extract was removed by rotary evaporation. The concentrate and the supernatant of the extract were freeze-dried and weighed. Each extraction was conducted in duplicate. The stock solution was used for total phenolic compound (TPC) content analysis, antioxidant capacity evaluation, and antimicrobial property determination.

For the determination of the TPC content, the Folin–Ciocalteu TPC content assay was used. The method used was adapted from Ainsworth and Gillespie [42]. One millilitre of the sample and (or) standard solution was added to each cuvette (10 × 45 mm, 3 mL), followed by 5000 µL 10% (*v*/*v*) Folin–Ciocalteu reagent in super distilled water. Further, 4000 µL 7.5% (*v*/*v*) Na_2_CO_3_ in super distilled water was added to the centrifuge tubes. The mixture was incubated for 60 min, and the absorbance was measured at 765 nm using a Genesys-10 UV/VIS spectrophotometer (Thermo Spectronic, Rochester, NY, USA). The data were expressed as gallic acid equivalents (GAE) mg 100 g^−1^ d.w. (dry weight). The antioxidant (DPPH° scavenging) capacity of the algal extracts was determined by the method of Brand-Williams et al. [43] with modifications as described elsewhere [44]. Twenty microlitres of extract was allowed to react with 2 mL of DPPH° solution for 30 min in the dark. The decrease in absorbance was measured at 515 nm using a Genesys-10 UV/VIS spectrophotometer (Thermo Spectronic, Rochester, NY, USA). The ferric reducing antioxidant power (FRAP) assay was carried out by the method of Benzie and Strain [45] with some modifications [46]. Two millilitres of freshly prepared FRAP working solution and 20 µL of extract were mixed in a cuvette with a 1-cm path length and incubated for 30 min at ambient temperature. The change in absorbance due to the reduction of the ferric-tripyridyltriazine (Fe III-TPTZ) complex by the antioxidants present in the samples was measured at 593 nm using a Genesys-10 UV/VIS spectrophotometer. 

### 2.4. Measurement of the Algae Colour Chromaticity Parameters

The colour coordinates of the lyophilized algae and their extracts were evaluated on the surface using a CIE L*a*b* system (CromaMeter CR-400, Konica Minolta, Marunouchi, Tokyo Japan) [31,44].

### 2.5. Evaluation of the Antimicrobial Activity of the Algal Extract Samples

All algal samples were assessed for their antimicrobial activities against a variety of pathogenic and opportunistic bacterial strains (*Escherichia coli*, *Klebsiella pneumoniae*, *Salmonella enterica*, *Cronobacter sakazakii*, *Acinetobacter baumannii*, *Pseudomonas aeruginosa*, *Staphylococcus aureus*, *S. haemolyticus*, *Bacillus subtilis,* and *Streptococcus mutans*) by using the agar well diffusion method and in liquid medium. 

For the agar well diffusion assay, suspensions of 0.5 McFarland standard of each pathogenic bacterial strain were inoculated onto the surface of cooled Mueller–Hinton agar (Oxoid, Basingstoke, UK) using sterile cotton swabs. Wells 6 mm in diameter were punched in the agar and filled with 50 µL of the algal extract. The antimicrobial activities against the tested bacteria were established by measuring the inhibition zone diameters (mm). The experiments were repeated three times, and the average diameter of the inhibition zones was calculated. 

To evaluate the antimicrobial activity of the algal extracts in liquid medium, the algal samples were diluted 1:3 (*v*/*v*) with physiological solution. Then, to the different concentrations of extracts (10 µL, 50 µL, 100 µL, and 500 µL), 10 µL of the pathogenic and opportunistic bacterial strains, cultured in a selective medium, was added and incubated at 35 °C for 24 h. After incubation, the viable pathogenic and opportunistic bacterial strains in algal extract solution were controlled by plating them on selective medium. The results were interpreted as (−) if the pathogens did not grow on selective medium and (+) if the pathogens grew on selective medium. Experiments were performed in triplicate.

### 2.6. Analysis of Micro- and Macroelements in Algal Extract Samples

Analysis of micro- and macroelements in algal extract samples was performed by inductively coupled plasma mass spectrometry (ICP-MS) according to a published method [47].

### 2.7. Statistical Analysis

Extract preparation of algal samples was performed in duplicate, while all analytical experiments were carried out in triplicate. The calculated mean values, using the statistical package SPSS for Windows (Ver.15.0, SPSS, Chicago, IL, USA), were compared using Duncan’s multiple range test with significance defined at *p* ≤ 0.05. A linear Pearson’s correlation was used to quantify the strength of the relationship between the variables. The results were recognised as statistically significant at *p* ≤ 0.05.

## 3. Results and Discussion

### 3.1. Total Carotenoid and Chlorophyll a and b Contents in Algal Samples

**Figure 2:**
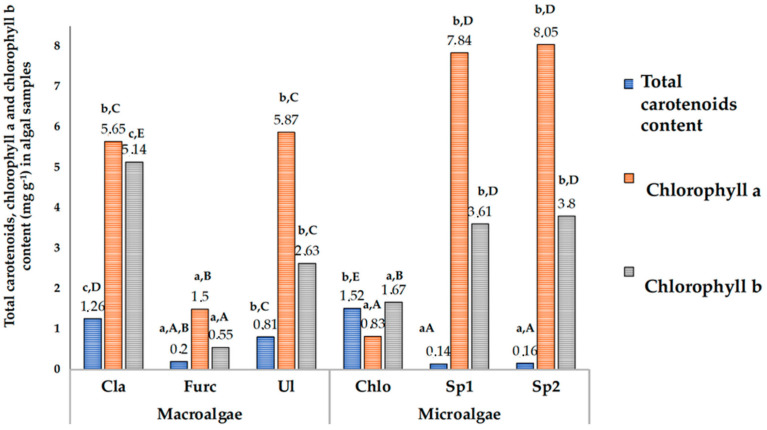
Total carotene, chlorophyll a, and chlorophyll b content (mg g^−1^) in algal samples (Cla, *Cladophora rupestris*; Furc, *Furcellaria lumbricalis*; Ul, *Ulva intestinalis*; Chlo, *Chlorella vulgaris*; Sp1, Spirulina (*Arthrospira platensis*) from University of Texas; Sp2, Spirulina (Ltd. “Spila“). a–c for the same analytical parameters, in macro- and microalgae groups, means with different letters are significantly different (*p* ≤ 0.05). A–E for the same analytical parameters, in all algal samples, means with different letters are significantly different (*p* ≤ 0.05)).

Total carotenoid and chlorophyll a and b contents in the algal samples (d.w.) are given in Figure 2. 

In a comparison of macroalgae samples, the highest TCC was found in *C. rupestris* samples (1.26 mg/g). In *U. intestinalis* and *F. lumbricalis* samples, the TCC was 1.6- and 6.3-times lower, respectively. With respect to the TCC in microalgal samples, *C. vulgaris* showed the highest concentration (1.52 mg/g), which was an average of 10.1-times higher than that in both the tested Spirulina (Sp1 and Sp2) samples. However, the chlorophyll a and b concentrations in *C. vulgaris* samples were, on average, 89.6 and 55.0% lower than those in both the tested Spirulina samples. The green microalgae *C. vulgaris* contains high levels of carotenoids [48]. The main carotenoids in *C. vulgaris* are lutein and β-carotene [49,50]. These pigments are essential for the photosynthetic system of microalgae [48]. Additionally, carotenoids are associated with chlorophyll in the thylakoid membrane of chloroplasts, where they function to protect chlorophyll molecules from degradation [51]. *A. platensis* is also good source of carotenoids [52] with various desirable properties [53,54]. However, *A. platensis* is also a good source of chlorophylls [55]. Of all the tested algal samples, the microalgae *C. vulgaris* was the best source of carotenoids, followed by the macroalgae *C. rupestris*, in which the TCC was lower by 17.1% in comparison with that of *C. vulgaris*. In addition, when the TCC in algal samples was increased, the chlorophyll a concentration was reduced, and a moderate negative correlation between the chlorophyll a and TCC in algal samples was found (r = −0.4644). Moreover, a strong positive correlation between the chlorophyll a and b concentrations was established (r = 0.7604). However, a very weak positive correlation was found between the chlorophyll b and TCC (r = 0.1065).

**Figure 3:**
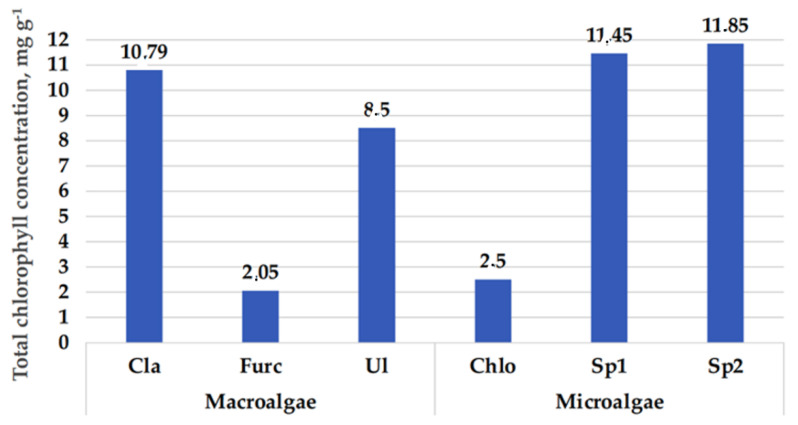
Total chlorophyll concentration (mg g^−1^) in algal samples (Cla, *Cladophora rupestris*; Furc, *Furcellaria lumbricalis*; Ul, *Ulva intestinalis*; Chlo, *Chlorella vulgaris*; Sp1, Spirulina (*Arthrospira platensis*) from University of Texas; Sp2, Spirulina (Ltd. “Spila“).

In a comparison of chlorophyll a and b concentrations in macroalgal samples, in all the tested macroalgae samples, the predominant form of chlorophyll was chlorophyll a (in comparison with chlorophyll b, the chlorophyll a content in *C. rupestris*, *F. lumbricalis,* and *U. intestinalis* samples was higher by 9.0, 63.3, and 55.2%, respectively). The highest total chlorophyl content was found in *Cladophora rupestris* (in comparison macroalgae samples) as well as in both the tested Spirulina samples (in comparison microalgae samples) (Figure 3). *C. vulgaris* contains the green pigment (chlorophyll) and carotenoids [19], and the composition of pigment in *Spirulina platensis* is complex and could include chlorophyll, xanthophylls, phycocyanin, and carotenoids consisting of myxoxanthophyll, beta carotene, and zeaxanthin [56]. In this study, a limited number of the pigments was analysed; however, according to results obtained, the highest total chlorophyl content was found in *Cladophora rupestris* (in comparison macroalgae samples) and in both the tested Spirulina samples (in comparison microalgae samples), and the highest TCC was established in *Chlorella vulgaris* and *Chlorella vulgaris*. Additionally, it should be pointed out that extraction method has an influence on pigments concentration in the extract [57]; for this reason, it is very difficult to compare results from different studies.

There is a broad application of chlorophylls because its intensive green colour is gaining importance as a food additive. European Food Safety Authority (EFSA) published maximum permission levels (MPLs) of chlorophylls as food additives for use in foods [58]. Chlorophylls are authorised food additives in the EU at quantum satis (QS) in 56 food categories [58]. Chlorophyll a is usually present in foods at a concentration 2–3-times higher than chlorophyll b. Chlorophyll, as a food ingredient, increases its biological functions [59].

In addition, carotenoids are naturally occurring plant pigments that are responsible for the colours of different red, green, yellow, and orange fruits, vegetables, and algae. While the most studied carotenoid is beta-carotene, other carotenoids, such as lycopene, are now receiving much attention due to their higher antioxidant activity and organ-specific functionality in comparison with beta-carotene. Carotenoids cannot be synthesized by mammals and, therefore, have to be obtained from food/feed sources or in a form of dietary and (or) feed supplements [60,61]. A group acceptable daily intake (ADI) of 0–5 mg kg^−1^ bw for β-carotene, β-apo-8′-carotenal, and β-apo-8′-carotenoic acid methyl and ethyl esters was established [62]. Optimal carotenoids intake is related to reduced risks of several diseases [62]. In addition, carotenoids are used in the feed industry to improve animal health and animal based products quality [63].

### 3.2. Antioxidant Characteristics and Chromaticity Parameters of the Algal Samples

**Figure 4:**
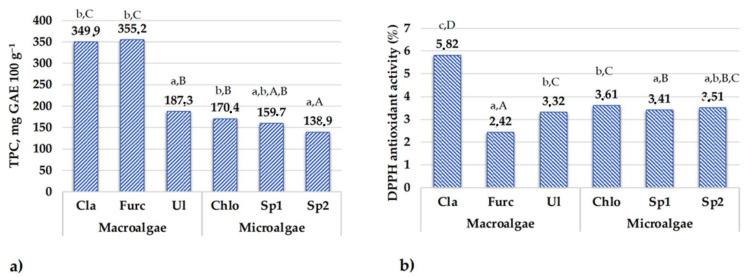
(**a**) Total phenolic compound content (mg GAE 100 g^−^) and (**b**) DPPH antioxidant activity (%) of the algae extracts (Cla, *Cladophora rupestris*; Ul, *Ulva intestinalis*; Furc, *Furcellaria lumbricalis*; Chlo, *Chlorella vulgaris*; Sp1, Spirulina (*Arthrospira platensis*) multiplied in the laboratory; Sp2, Spirulina (Ltd. “Spila“); TPC, total phenolic compounds content; GAE, gallic acid equivalents; DPPH, 1,1-diphenyl-2-picrylhydrazyl; a–c for the same analytical parameters, in macro- and microalgae groups, means with different letters are significantly different (*p* ≤ 0.05). A–D for the same analytical parameters, in all algal samples, means with different letters are significantly different (*p* ≤ 0.05)).

The TPC content and DPPH antioxidant activity of the different specimen of algae extracts are shown in Figure 4a,b, respectively, and colour coordinates of the lyophilized algae and their extracts are given in Table 1. 

In a comparison of macroalgae extract samples, *C. rupestris* and *F. lumbricalis* showed the highest TPC content (on average, 352.6 mg GAE 100 g^−1^), and in *U. intestinalis,* the TPC content was an average of 53.1% lower. A weak positive correlation between the TPC content in macroalgae samples and DPPH antioxidant activity was found (r = 0.2419); however, the *C. rupestris* extract had significantly higher DPPH antioxidant activity (5.82%) than the *U. intestinalis* and *F. lumbricalis* extracts. Extracts of *U. intestinalis* and *F. lumbricalis* showed an average of 1.8- and 2.4-times lower DPPH antioxidant activity, respectively, in comparison with *C. rupestris* extract. Moderate positive correlations between the TPC content and redness (a*) in both lyophilized samples and extracts were found (r = 0.7067 and r = 0.8069, respectively). Additionally, negative correlations between the TPC content and yellowness (b*) in both lyophilized samples and extracts were established (r = −0.9787 and r = −0.9813, respectively). The DPPH antioxidant activity of the macroalgae samples was related to the total carotenoid content in samples, and a very strong positive correlation between the above-mentioned parameters was found (r = 0.9372). In addition, moderate and very strong positive correlations between the DPPH antioxidant activity and chlorophyll a and b contents in macroalgae were established (r = 0.6731 and r = 0.9771, respectively). Antioxidant activities reduces oxidation processes [64], and both scavenging and antioxidant activities are related with the content of polyphenolic compounds [65]. Coloured compounds, in many cases, lead to higher antioxidant properties of the product and (or) extract; however, specific of the antioxidant properties is related with the specific of phenolic compounds profile composition. From this point of view, relation of colour coordinates with parameters of the antioxidant properties could be very important for the further analysis and (or) extraction methods development, to select which compounds, in relation with their chromaticity parameters, can have a higher antioxidant potential.

Santoso et al. [66] and Wang et al. [67] reported that synergistic effects among the different substances on the TPC concentration in algae should also be considered. Messyasz et al. [68] reported that there is a strong correlation between the antioxidant activity of the sample, and the TPC content in *Cladophora* and *Cladophora* extracts could be a promising source of pharmaceuticals [69] as well as for the food industry [8]. Overall, freshwater species are examined more often in comparison with marine ones [8]. Additionally, it was published that *U. intestinalis* possesses antioxidant and cytotoxic activity [70], and the antioxidant characteristics of its extracts depend on the extractant used: the antioxidant activity of dichloromethane, ethanol, methanol, and hexane extracts was 87.54, 31.9, 22.6, and 22.5% [71]. However, Farasat et al. [72] reported that methanolic extracts of *U. intestinalis* showed the highest DPPH scavenging activity (48% inhibition) and a lower IC50 value of 2.32 mg/mL. Tepe et al. [73] reported that polar extracts showed stronger antioxidant activity than non-polar extracts prepared from *Salvia tomentosa*, and according to Naczk and Shahidi [74], the polarity of the solvent has a significant influence on increasing the solubility of phenols. Moreover, it was published that the TPC content of *U. intestinalis* extracts ranged from, on average, 54.4 to 197 mg GAE/g, and a higher TPC content resulted in a higher antioxidant activity [71]. Naseri et al. [75] showed that *F. lumbricalis* had the highest TPC, followed by *C. crispus* and *S. crispate*, and these values were higher than those obtained for *Palmaria palmata*, *Chondrus crispus, Meristotheca papulosa,* and *Sarcodiotheca gaudichaudi* (Rhodophyta) [76,77].

In a comparison of the TPC content in microalgal samples, in *A. platensis* samples, TPC was, on average, 149.3 mg GAE 100 g^−1^ and in *C. vulgaris* extracts, on average, 12.4% higher. Similar tendencies in the DPPH antioxidant activity of the microalgae extracts were found, and the highest DPPH antioxidant activity was established in *C. vulgaris* extracts (3.61%). A moderate positive correlation was established between the TPC content and DPPH antioxidant activity of microalgae extracts (r = 0.5979). Positive and negative moderate correlations, respectively, between the TPC content and redness (a*) of lyophilized samples and extracts of microalgae were found (r = 0.5000 and r = −0.6708, respectively). In addition, positive strong correlations between the TPC content and yellowness (b*) were established in both lyophilized samples and extracts (r = 0.7797 and r = 0.7598, respectively). As well, a strong positive correlation between the TPC content in microalgae extracts and lightness (L*) was found (r = 0.7389). DPPH antioxidant activity and the TPC content of the microalgae samples was related to the total carotenoid content in samples, as very moderate positive correlations between the above-mentioned parameters were found (r = 0.6191 and r = 0.6439, respectively). Additionally, moderate negative correlations between the microalgae TPC content and chlorophyll a and b were established (r = −0.5733 and r = −0.5157, respectively). A negative moderate correlation between the DPPH antioxidant activity and chlorophyll a was found (r = −0.5708).

Microalgae contain high levels of phenolic compounds, which contribute to the antioxidant activity of their extracts [78]. Agregan et al. [79] reported that the antioxidant capacity of *Spirulina* extract is higher than that of *C. vulgaris*. However, in our study, different tendencies were established, and this can be explained by the various techniques used for extract preparation. Finally, both microalgae showed antioxidant potential, which could be very promising for further microalgae extract applications.

In a comparison of all the tested extracts, *C. rupestris* and *F. lumbricalis* showed the highest TPC content, and the highest DPPH antioxidant activity was found in *C. rupestris* extracts.

### 3.3. Antimicrobial Activity of the Algal Extract Samples

**Figure 5:**
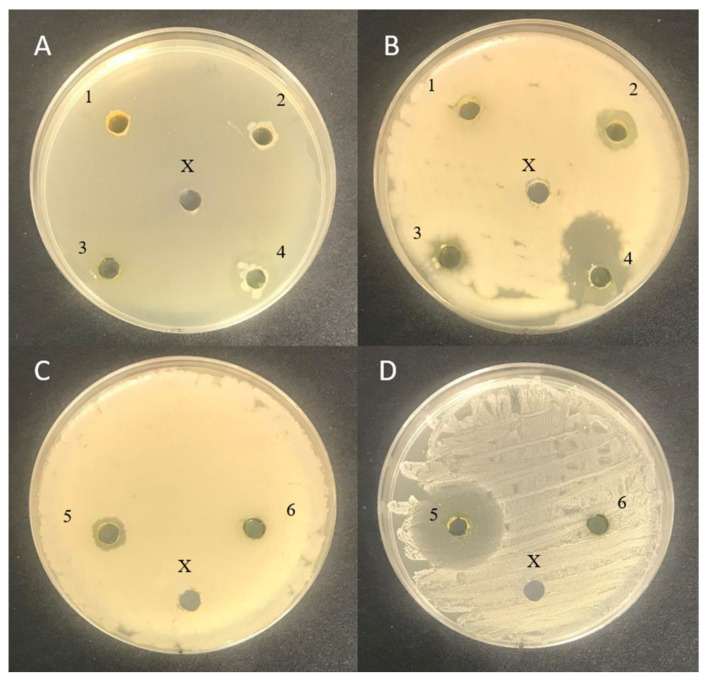
Antimicrobial activity of the algal extract samples assessed by using the agar well diffusion method (**A**) *Streptococcus mutans*; (**B**) *Bacillus subtilis*; (**C**) *Bacillus subtilis*; (**D**) *Staphylococcus haemolyticus*; 1—Spirulina (*Arthrospira platensis*) from University of Texas; 2—*Chlorella vulgaris*; 3—*Cladophora rupestris*; 4—*Furcellaria lumbricalis*; 5—*Ulva intestinalis*; 6—Spirulina (Ltd. “Spila“); X—control (physiological solution).

Antimicrobial activity results of the algal extract samples assessed by using the agar well diffusion and liquid medium methods are given in Table 2 and Figure 5. None of the tested macro- and microalgae extracts possessed antimicrobial activities in liquid medium; however, by using the agar well diffusion method, *A. platensis* samples obtained from University of Texas extracts were shown to inhibit *S. haemolyticus* (diameter of inhibition zone 28.3 mm). In addition, all the tested macroalgae extracts (*C. rupestris*, *F. lumbricalis,* and *U. intestinalis*) showed inhibitory properties against *B. subtilis* (with inhibition zone diameters of 12.0, 8.0, and 17.0 mm, respectively). Moreover, *B. subtilis* was inhibited by *A. platensis* samples obtained from University of Texas extracts (diameter of inhibition zone: 10.1 mm). In addition, *U. intestinalis* extracts inhibited *S. mutans* (14.2 mm).

It was reported that green, red, and brown algae showed antifungal, antibacterial, cytostatic, antiviral, anthelmintic, etc., properties [80,81,82], and algae extracts could inhibit bacteria, yeast, and fungi [35,83,84,85,86]. This may be related to the different algae metabolites that possess antimicrobial properties [87,88,89]. It was reported that algae extracts displayed antimicrobial properties against gram-positive and -negative bacterial strains [90,91,92,93]. Some of the studies hypothesized that organisms in stressed environments might develop compounds with antimicrobial properties [94,95,96,97,98,99,100], and this may be applied to *U. intestinalis* [101,102].

Srikong et al. [71] demonstrated that *U. intestinalis* extracts inhibited gram-positive bacteria; however, extracts do not show inhibitory properties against gram-negative bacteria. In addition, it was reported that the antimicrobial activity of the algae extracts depended on the extractant used [71,83,103]. However, differences in antimicrobial characteristics could be due to differences in the production of antimicrobial compounds, which are related to seasonal variations [104,105]. Moreover, differences in extraction protocols [106,107,108] and differences in the stage of active growth, etc. [109,110], could also result in differences in antimicrobial activity. The ability of extracts to inhibit gram-positive and not gram-negative bacteria may be associated with differences in permeability barriers [71] because in gram-negative species, the outer membrane is a barrier that does not allow the tested compounds to pass [111]. Phenolics from marine algae attack the cell walls and cell membranes of the pathogens [71]. Moreover, fatty acids in algae (myristic acid, palmitic acid, and *cis*- 8-octadecanoic acid) can inhibit bacteria [35,112,113]. It was reported that the antimicrobial activity of *Cladophora* extract can be attributed to the presence of fatty acids [37,114,115,116]. Stabili et al. [116] reported that α-linolenic acid could be involved in antibacterial activity. In addition, Laungsuwon and Chulalaksananukul [115] reported that *Cladophora* extracts also contained other antimicrobial compounds (alkanes, phenols, imidazole, 2-amino-5-[(2-carboxy)vinyl]-, 2,4-di-tertbutylphenol, and dihydroactinidiolide) as well as thymol [32]. It was also reported that methanolic *Spirulina* extract metabolites inhibited both gram-positive and gram-negative pathogens [117]. In our study, *A. platensis* extracts showed inhibitory properties against *S. haemolyticus* and *B. subtilis* strains, and *B. subtilis* was inhibited by *C. rupestris*, *F. lumbricalis,* and *U. intestinalis* macroalgae extracts. *S. haemolyticus* can be found in respiratory and gastrointestinal mucosal membranes, anterior nares, ear canals, inguinal areas, etc., of humans and animals [118]. Among coagulase-negative staphylococci, *S. haemolyticus* is only second to *S. epidermidis* in causing bloodstream infections [119]. *S. haemolyticus* is a very dangerous pathogen because of the extreme plasticity of its genome, which leads to its multi-drug resistance characteristics [120]. Additionally, *S. haemolyticus* can cause gangrenous mastitis in dairy cows [121]. From this point of view, extracts of selected A. platensis varieties could be very promising ingredients for their desirable antimicrobial properties against *S. haemolyticus* for human drugs as well as veterinary drug preparations. The *Bacillus subtilis* inhibitory properties of extracts of *A. platensis* and macroalgae *C. rupestris*, *F. lumbricalis,* and *U. intestinalis* could be applied to the reduction of cereal product spoilage. Although the spoilage of bakery products is mainly due to moulds, the roping of the bread, caused by *Bacillus* sp., especially *B. subtilis*, is also a very large economical challenge [122,123]. Extracts of *U. intestinalis* showed inhibitory properties against *S. mutans*, an anaerobic gram-positive bacterium [124], which causes dental caries [125]. *S. mutans* secretes glycosyltransferases, which synthesize intracellular and extracellular polysaccharides [126]. It was reported that water-insoluble glucans induce the adherence of oral bacteria to the tooth surface, thereby promoting plaque and cavity formation [126,127]. To avoid cavities, sodium fluoride and chlorhexidine have been used [128,129]; however, these substances can lead to resistance of oral-related pathogens in the oral microbiome [130]. From this point of view, the inhibitory properties of the *U. intestinalis* extracts against *S. mutans* are paramount, and they can be recommended as natural ingredients for oral health improvement.

### 3.4. Micro- and Macroelement Concentrations in Algal Samples

Micro- and macroelement concentrations in algal extract samples are shown in Table 3.

Sodium (Na) concentration was 2.1-times higher in *U. intestinalis* than in *C. rupestris,* and *F. lumbricalis*. *U. intestinalis* extracts also showed the highest magnesium (Mg) concentration (on average, 1.9- and 1.3-times higher than in *C. rupestris* and *F. lumbricalis*, respectively). The bioaccessibility of Mg varies between algae species, and it was reported that the Mg bioaccessibility of *Ulva australis* (formerly *Ulva pertusa*) (Chlorophyta), *Saccharina japonica* (formerly *Laminaria japonica*) (Phaeophyceae), and *Gloiopeltis furcata* (Rhodophyta) is 41.8, 60.8, and 72.5%, respectively [131]. The highest concentration of potassium (K) was established in *C. rupestris* extract (975 mg kg^−1^ d.m.), and in *F. lumbricalis* and *U. intestinalis* extracts, the K concentration was, on average, 2.2- and 2.9-times lower, respectively. In *C. rupestris*, *F. lumbricalis* and *U. intestinalis* extracts, the Na/K ratio was 0.23, 0.62, and 1.53, respectively. According to World Health Organization (WHO) recommendations, Na intake should not to exceed 3.5 g/day [132]. The lowest content of calcium (Ca) was found in *U. intestinalis* extracts (on average, 2.1-times lower compared with *C. rupestris* extract and, on average, 3.6-times lower compared with *F. lumbricalis*). According to Waheed et al. [133], insufficient Ca consumption is major challenge for public health these days; for this reason, new natural sources of Ca are sought for inclusion human and (or) animal diets. In a comparison of the macroelement concentrations in macroalgae extracts with those in fresh macroalgae samples evaluated in our previous studies [31], in all cases, the macroelement concentrations in extracts were increased: in *C. rupestris* extracts, the Na, Mg, K, and Ca concentrations increased, on average, by factors of 50.1, 50.2, 70.7, and 8.9, respectively.

In *F. lumbricalis* extracts, the Na, Mg, K, and Ca concentrations increased, on average, by factors of 53.1, 21.7, 20.5, and 6.2, respectively, and in *U. intestinalis* extracts, the Na, Mg, K, and Ca concentrations increased, on average, by factors of 29.0, 16.1, 43.6, and 1.6, respectively. From this point of view, the extraction technology used in this study for macroalgae pre-treatment could be an appropriate method to concentrate desirable macroelements of the algal samples for further use in the nutraceutical and pharmaceutical industries. 

Regarding macroelements in microalgae algae extract samples, the highest sodium (Na) concentration was found in Spirulina (Ltd. “Spila“) extracts (Sp1) (458 mg kg^−1^ d.m.), and, in comparison with Spirulina (*A. platensis*) obtained from the University of Texas extracts (Sp2), the Na concentration in Sp1 extracts was, on average, 3.4-times lower. Additionally, Sp2 extracts showed the highest magnesium (Mg) concentration (on average, 3.3- and 4.4-times higher than that in Sp1 and *C. vulgaris* extracts, respectively). The highest concentration of potassium (K) was also established in Sp2 extract (950 mg kg^−1^ d.m.), and in Sp1 and *C. vulgaris* extracts, the K concentration was, on average, 8.6- and 12.3-times lower, respectively. In *C. rupestris*, the Na/K Sp1 ratio in Sp2 extracts was 0.35, 1.2, and 0.48, respectively. The lowest content of calcium (Ca) in *C. vulgaris* extracts was found (on average, to be 3.0-times lower compared with Sp1 extract and, on average, 12.3-times lower compared with Sp2 extracts). It was reported that macroelements in Spirulina ranged in the order K > Ca > P > Na > Mg and in *Chlorella,* in the order K > P > Ca > Na > Mg; moreover, a higher content of Ca was found in Spirulina than in *Chlorella*, but a higher concentration of P was established in *Chlorella* in comparison with Spirulina [134].

In a comparison of essential microelement concentrations in different macroalgae specimens, the highest concentration of manganese (Mn), iron (Fe), cobalt (Co), and nickel (Ni) was found in *C. rupestris* extracts (on average, 7.41 mg kg^−1^, 4.34 mg kg^−1^, 0.064 mg kg^−1^, and 0.301 mg kg^−1^, respectively). *U. intestinalis* showed the highest concentration of copper (Cu) (on average, 0.202 mg kg^−1^). In all analysed macroalgae samples, the concentration of selenium (Se) was, on average, 0.002 mg kg^−1^. However, the highest concentration of cromium (Cr) was, on average, 18.0- and 1.8-times higher in *F. lumbricalis* extracts in comparison with *C. rupestris* and *U. intestinalis* extracts. In contrast with our previous studies [31], zinc (Zn), iodine (I), and phosphorus (P) did not remain in macroalgae extracts; however, their concentrations in fresh macroalgae samples were 16.5 mg kg^−1^, 199.0 mg kg^−1^, and 0.984 mg kg^−1^, respectively, in *C. rupestris*; 26.5 mg kg^−1^, 49.5 mg kg^−1^, and 1.56 mg kg^−1^, respectively, in *F. lumbricalis;* and 38.7 mg kg^−1^, 22.8 mg kg^−1^, and 1.93 mg kg^−1^, respectively, in *U. intestinalis* [31]. Most of the essential microelement concentrations in extracts decreased in comparison with those in fresh macroalgae samples (except those of manganese (Mn), which increased by factors of 10.6, 3.4, and 12.1 in *C. rupestris*, *U. intestinali, s* and *F. lumbricalis* extracts, respectively, and iron (Fe), which increased by factors of 4.2 and 9.3, respectively, in *C. rupestris* and *F. lumbricalis* extracts). Chromium (Cr) decreased by factors of 227.0, 495.7, and 0.24, in *C. rupestris*, *U. intestinalis,* and *F. lumbricalis* extracts, respectively; Fe decreased by a factor of 1.2 in *U. intestinalis* extracts; Co decreased by factors of 21.6, 65.3, and 125.3 in *C. rupestris*, *U. intestinalis,* and *F. lumbricalis* extracts, respectively; Ni decreased by factors of 19.9, 320.0, and 244.0 in *C. rupestris*, *U. intestinalis,* and *F. lumbricalis* extracts, respectively; Cu decreased by factors of 97.8, 74.8, and 2625 in *C. rupestris*, *U. intestinalis,* and *F. lumbricalis* extracts, respectively, and Se decreased by factors of 58.0, 100.0, and 200.0 in *C. rupestris*, *U. intestinalis,* and *F. lumbricalis* extracts, respectively. The WHO recommends a Se intake of 40 µg day^−1^ for men and 30 µg day^−1^ for women [135]. The European Food Safety Authority (EFSA) for adults proposed intake of Mn is 3 mg day^−1^ [136]. Data on the potential toxicity of Co are scarce [137]. According to the WHO Regional Office for Europe [138], Ni concentrations corresponding to an excess lifetime risk of 1:10,000, 1:100,000, and 1:1,000,000 are approximately 250, 25, and 2.5 ng (m^3^)^−1^. Non-desirable changes in extracts were observed in accordance with I reductions; however, the I intakes recommended by the WHO, EFSA, and U.S. Food and Nutrition Board Institute of Medicine are 100–150 μg day^−1^ [132], 150 μg day^−1^, and 1100 μg day^−1^. Algae may be a good source of Fe; however, literature data regarding micro- and macroelement concentrations in algae extracts are scarce. We found that in *C. rupestris* and *F. lumbricalis* extracts, the Fe concentration increased by factors of 4.2 and 9.3, respectively. García-Casal et al. reported that *Sargassum spp*. (Phaeophyceae) contained 156.9 mg 100 g^−1^ d.w. of Fe [139]. The EFSA reported that the adequate intake (AI) for Cr is not appropriate, and for Cu, the proposed AI is 1.6 mg day^−1^ for men and 1.3 mg day^−1^ for women [136]. The average requirements for Zn are 7.3 and 5.5 mg day^−1^ for men and women, respectively [140], and the U.S. recommended daily intake is 11 mg day^−1^ for men and 8 mg day^−1^ for women [141]. Additionally, the WHO recommendations are that adults should not exceed a Zn intake of 45 mg day^−1^ (World Health Organization, 1996). According to the EFSA, in European countries, P intake is estimated, on average, at 1000–1500 mg day^−1^ [140].

In a comparison of essential microelement concentrations in microalgae extracts, the highest concentrations of most of the determined essential microelements (Cr, Mn, Fe, Co, Ni, and Se) were found in Sp2 extracts (on average, 0.056 mg kg^−1^, 0.333 mg kg^−1^, 4.73 mg kg^−1^, 0.040 mg kg^−1^, 0.020 mg kg^−1^, and 0.004 mg kg^−1^, respectively). Both *C. vulgaris* and Sp2 extracts showed the highest concentrations of copper (Cu) (on average, 0.074 mg kg^−1^). It was reported that the average concentration of essential microelements decreased in the order Fe > Mn > Zn > Cu > Cr > Co > Mo = Se in Spirulina and Fe > Mn > Zn > Cu > Cr > Mo > Se > Co in *Chlorella* [134]. 

In a comparison of non-essential microelements in macroalgae extracts, the highest arsenic (As), molybdenum (Mo), stibium (Sb), and lithium (Li) concentrations were found in *U. intestinalis* extracts (0.437 mg kg^−1^, 0.007 mg kg^−1^, 0.163 mg kg^−1^, and 0.047 mg kg^−1^, respectively). The lead (Pb) concentration in all the tested extracts was, on average 0.002 mg kg^−1^. *C. rupestris* extracts showed the highest concentration of rubidium (Rb), on average, higher than that in *F. lumbricalis* and *U. intestinalis* extracts by factors of 2.0 and 3.6, respectively. The vanadium (V) concentration in all the tested macroalgae extract was, on average, 0.005 mg kg^−1^. The highest concentration of strontium (Sr), silver (Ag), and aluminium (Al) was found in *F. lumbricalis* extracts (0.621 mg kg^−1^, 0.098 mg kg^−1^, and 1.14 mg kg^−1^, respectively). In comparison with *U. intestinalis* extracts, the barium (Ba) concentration in *C. rupestris* and *F. lumbricalis* extracts was higher by an average factor of 4. Titanium (Ti) and cadmium (Cd) were not found in macroalgae samples, and caesium (Cs) was only established in *F. lumbricalis* extract (0.001 mg kg^−1^). In a comparison of extracts and fresh algae samples (Tolpeznikaite et al., 2021), gallium (Ga), beryllium (Be), tin (Sn), mercury (Hg), boron (B), titanium (Ti), and cadmium (Cd) did not remain in extracts, and molybdenum (Mo) was not obtained in *C. rupestris* extracts, nor was caesium (Cs) in *C. rupestris* and *U. intestinalis* extracts. Most of the non-essential microelement concentrations decreased in extracts in comparison with fresh macroalgae samples (except that of aluminum (Al), which increased by a factor of 7.6 in *F. lumbricalis* extracts): arsenic (As) decreased by factors of 11.0, 9.9, and 22.0 in *C. rupestris*, *U. intestinalis,* and *F. lumbricalis* extracts, respectively; V decreased by factors of 291.7, 557.5, and 297.5 in *C. rupestris*, *U. intestinalis,* and *F. lumbricalis* extracts, respectively; rubidium (Rb) decreased by factors of 18.4, 41.2, and 54.8 in *C. rupestris*, *U. intestinalis,* and *F. lumbricalis* extracts, respectively; strontium (Sr) decreased by factors of 341.7, 746.9, and 331.7 in *C. rupestris*, *U. intestinalis,* and *F. lumbricalis* extracts, respectively; Mo decreased by factors of 35.7 and 62.5 in *U. intestinalis* and *F. lumbricalis* extracts, respectively; Ag decreased by factors of 13.9, 8.6, and 2.6 in *C. rupestris*, *U. intestinalis,* and *F. lumbricalis* extracts, respectively; Sb decreased by factors of 5.4, 1.5, and 50.0 in *C. rupestris*, *U. intestinalis,* and *F. lumbricalis* extracts, respectively; Cs decreased by a factor of 22.0 in *F. lumbricalis* extracts; Ba decreased by factors of 731.3, 2437.5, and 433.3 in *C. rupestris*, *U. intestinalis,* and *F. lumbricalis* extracts, respectively; Pb decreased by factors of 690.0, 760.0, and 128.3 in *C. rupestris*, *U. intestinalis,* and *F. lumbricalis* extracts, respectively; Al decreased by factors of 2.7 and 7.3 in *C. rupestris* and *U. intestinalis* extracts, respectively; and Li decreased by factors of 62.5, 16.1, and 17.4 in *C. rupestris*, *U. intestinalis,* and *F. lumbricalis* extracts, respectively.

The average consumption of the microelement V with food is 10–20 μg day^−1^ [140]. One of the main challenges associated with the safety of macroalgae consumption is its contamination with heavy metals, such as Al, Cd, Pb, Rb, Si, Sr, and Sn [142], which can lead to a public health risk [143]. Heavy metal control in macroalgae-based products must be included, and the different bio-absorption capacities of heavy metals should be taken into consideration [144]. The main challenge is associated with As (inorganic) contamination, which is categorized as a class I carcinogen, and other forms of As are categorized as potentially toxic [145]. Maximum concentrations for As in foodstuffs set from 0.10 to 0.3 mg kg^−1^; however, the maximum concentration of As in macroalgae is not regulated [146]. It was reported that the highest heavy metal concentrations in Baltic Sea macroalgae were Cd—1.41 μg g^−1^, Pb—10.50 μg g^−1^, Ni—5.13 μg g^−1^, Zn—223 μg g^−1^, Cu—19.50 μg g^−1^, and Cr—4.38 μg g^−1^ [147]. Żbikowski et al. reported that the anthropogenic impact of Cu, Pb, and Zn was observed in the case of *Cladophora sp.* and *Ulva* (formerly *Enteromorpha*) sp. (Chlorophyta) because of their ability to accumulate metal contaminants from seawater, and it was suggested that *Cladophora* sp. and *Ulva* sp. could be good biomonitors of Pb, Cu, and Zn concentrations in the Baltic Sea [29]. For Cd and Pb, there are maximum permitted levels (MPL) set for vegetables, but macroalgae are not mentioned [146]. For Cd, Pb, and Hg, there are MPL (3.0, 3.0, and 0.1 mg kg^−1^ wet weight, respectively) for food supplements (including algae). In addition, the European feed legislation sets MPL for undesirable compounds in feed stock (12% moisture content): for total As < 2 mg kg^−1^, for Cd 1 mg kg^−1^, for Pb 5 mg kg^−1^, and for Hg in fish feed and marine feed ingredients 0.1 and 0.2 mg kg^−1^, respectively [148].

In a comparison of non-essential microelements in microalgae extracts, similar tendencies to those of the essential microelements were found, and the highest concentrations of As, Rb, Sr, Mo, Ag, Sb, Ba, Al, and Li were found in Sp2 extracts (on average, 0.022 mg kg^−1^, 0.181 mg kg^−1^, 0.346 mg kg^−1^, 0.164 mg kg^−1^, 0.010 mg kg^−1^, 0.098 mg kg^−1^, 0.043 mg kg^−1^, 1.15 mg kg^−1^, and 0.070 mg kg^−1^, respectively). Additionally, in Sp2 extracts, on average, 0.001 mg kg^−1^ titanium (Ti) and cadmium (Cd) was found. In all the tested microalgae extracts, the lead (Pb) concentration was, on average, 0.001 mg kg^−1^, and caesium (Cs) was not found in microalgae samples. Vanadium (V) was established in *C. vulgaris* and Sp2 extracts (on average, 0.001 mg kg^−1^). The toxic element content in microalgae can vary, and this can be related to contamination in the environment from which the biomass was originally derived [149,150]. Some microalgae uptake toxic metals, and from this point of view, the chemical quality of the medium has a profound effect on the presence of contaminants in microalgae biomass [134]. 

Finally, to our knowledge, the present study is the first to analyse the levels of micro- and macroelements in micro- and macroalgae extracts and discuss the micro- and macroelement transition from fresh algae to extracts. It could be concluded from this study that the applied extraction method is a suitable technology for toxic metal decontamination of micro- and macroalgae. However, it should be pointed out that some of the desirable microelements are reduced during the extraction, and only the final products, according to their specific composition, could be applied in food, feed, nutraceutical, pharmaceutical, etc., preparation. In the interest of transparency and public clarity, the raw data and materials underlying these results are available in the Supplementary Materials S1 [raw data materials], following an ethical review confirming the study’s scientific integrity.

## 4. Conclusions

With respect to the TCC in macroalgal samples, the highest TCC was found in *C. rupestris* samples (1.26 mg/g), and in comparison with microalgal samples, *C. vulgaris* showed the highest concentration of TCC (1.52 mg/g). The highest total chlorophyl content was found in *Cladophora rupestris* (in comparison macroalgae samples) and in both the tested Spirulina samples. The highest TPC content was found in *C. rupestris* and *F. lumbricalis* extracts, and the highest DPPH antioxidant activity was shown in *C. rupestris* samples. In addition, Sp2 extracts inhibited *S. haemolyticus*; *C. rupestris*, *F. lumbricalis*, *U. intestinalis*, and Sp2 extracts inhibited *B. subtilis*; and *U. intestinalis* extracts inhibited *S. mutans*. In addition, in this study, relation of the colour coordinates with parameters of the antioxidant properties were established, and these results could be very promising for further analysis and (or) extraction methods development to select compounds with the highest antioxidant potential by their chromaticity parameters. This study showed that extraction is a suitable technology for toxic metal decontamination of micro- and macroalgae; however, it should be pointed out that some desirable microelements are reduced during the extraction process. Finally, algae extracts, according to their specific composition and characteristics (antimicrobial, antioxidant, and micro- and macroelement contents) could be applied in food, feed, nutraceutical, pharmaceutical, etc., preparation.

## Figures and Tables

**Table 1:** Colour coordinates (L*, a*, b*) of the lyophilized ground algae samples of different specimens and their extracts.

Algal Extract Sample		Colour Coordinates, NBS					
		Algal Extract Samples			Lyophilised Algal Samples		
		a*	b*	L*	a*	b*	L*
Macroalgae	Cla	−8.67 ± 0.19 b,C	16.21 ± 0.11 c,C	32.06 ± 0.25 a,A	−1.60 ± 0.02 b,E	5.09 ± 0.04 b,C	47.66 ± 0.20 a,C
	Furc	−5.49 ± 0.14 c,E	14.38 ± 0.13 a,A	50.19 ± 0.31 c,F	1.32 ± 0.03 c,F	4.49 ± 0.03 a,B	47.54 ± 0.15 a,C
	Ul	−9.68 ± 0.16 a,B	28.87 ± 0.16 f,F	42.04 ± 0.27 b,C	−3.52 ± 0.02 a,D	11.08 ± 0.10 c,D	47.75 ± 0.17 a,C
Microalgae	Chlo	−7.97 ± 0.12 b,D	23.08 ± 0.19 e,E	46.61 ± 0.25 b,D	−7.93 ± 0.11 a,A	27.29 ± 0.03 c,F	43.93 ± 0.19 b,B
	Sp1	−2.10 ± 0.09 c,F	21.93 ± 0.18 d,D	47.07 ± 0.19 c,E	−5.49 ± 0.04 c,C	14.38 ± 0.11 b,E	50.19 ± 0.23 c,D
	Sp2	−13.41 ± 0.14 a,A	15.47 ± 0.13 b,B	37.40 ± 0.21 a,B	−6.56 ± 0.05 b,B	1.83 ± 0.13 a,A	39.98 ± 0.18 a,A

Cla, *Cladophora rupestris*; Ul, *Ulva intestinalis*; Furc, *Furcellaria lumbricalis*; Chlo, *Chlorella vulgaris*; Sp1, Spirulina (*Arthrospira platensis*) multiplied in the laboratory; Sp2, Spirulina (Ltd. “Spila“); L*, lightness; a*, redness or −a*, greenness; b*, yellowness or −b*, blueness; NBS, National Bureau of Standards units; data are represented as means (n = 3, replicates of analysis) ± SD. a–f for the same analytical parameters, in macro- and microalgae groups, means with different letters are significantly different (*p* ≤ 0.05). A–F for the same analytical parameters, in all algal samples, means with different letters are significantly different (*p* ≤ 0.05).

**Table 2:** Antimicrobial activity of the algal extract samples assessed by using the agar well diffusion and liquid medium methods.

Algal Extract Sample	Pathogenic and Opportunistic Bacteria Strains									
	*Escherichia* *coli*	*Klebsiella pneumonia*	*Salmonella enterica*	*Cronobacter sakazakii*	*Acinetobacter baumani*	*Pseudomona aeruginosa*	*Staphylococcus aureus*	*Staphylococcus haemolyticus*	*Bacillus subtilis*	*Streptococcus mutans*
	Inhibition zones by using the agar well diffusion, mm									
Cla	nd	nd	nd	nd	nd	nd	nd	nd	12.0 ± 0.3	nd
Furc	nd	nd	nd	nd	nd	nd	nd	nd	8.0 ± 0.2	nd
Ul	nd	nd	nd	nd	nd	nd	nd	nd	17.0 ± 0.4	14.2 ± 0.2
Chlo	nd	nd	nd	nd	nd	nd	nd	nd	nd	nd
Sp1	nd	nd	nd	nd	nd	nd	nd	28.3 ± 0.7	10.1 ± 0.5	nd
Sp2	nd	nd	nd	nd	nd	nd	nd	nd	nd	nd
Antimicrobial activity of the algal extracts in liquid medium										
Concentration of algae extract 10 µL, concentration of pathogen 10 µL										
Cla	+	+	+	+	+	+	+	+	+	+
Furc	+	+	+	+	+	+	+	+	+	+
Ul	+	+	+	+	+	+	+	+	+	+
Chlo	+	+	+	+	+	+	+	+	+	+
Sp1	+	+	+	+	+	+	+	+	+	+
Sp2	+	+	+	+	+	+	+	+	+	+
Concentration of algae extract 50 µL, concentration of pathogen 10 µL										
Cla	+	+	+	+	+	+	+	+	+	+
Furc	+	+	+	+	+	+	+	+	+	+
Ul	+	+	+	+	+	+	+	+	+	+
Chlo	+	+	+	+	+	+	+	+	+	+
Sp1	+	+	+	+	+	+	+	+	+	+
Sp2	+	+	+	+	+	+	+	+	+	+
Concentration of algae extract 100 µL, concentration of pathogen 10 µL										
Cla	+	+	+	+	+	+	+	+	+	+
Furc	+	+	+	+	+	+	+	+	+	+
Ul	+	+	+	+	+	+	+	+	+	+
Chlo	+	+	+	+	+	+	+	+	+	+
Sp1	+	+	+	+	+	+	+	+	+	+
Sp2	+	+	+	+	+	+	+	+	+	+
Concentration of algae extract 500 µL, concentration of pathogen 10 µL										
Cla	+	+	+	+	+	+	+	+	+	+
Furc	+	+	+	+	+	+	+	+	+	+
Ul	+	+	+	+	+	+	+	+	+	+
Chlo	+	+	+	+	+	+	+	+	+	+
Sp1	+	+	+	+	+	+	+	+	+	+
Sp2	+	+	+	+	+	+	+	+	+	+
Pathogen control										
Pathogen	+	+	+	+	+	+	+	+	+	+

Interpretation of results: negative (−) means the pathogens did not grow on the selective culture medium; positive (+) means the pathogens grew on the selective culture medium; nd - not determined. Value in brackets is the algae extract concentration in 10 mL of liquid media. Cla, *Cladophora rupestris*; Ul, *Ulva intestinalis*; Furc, *Furcellaria lumbricalis*; Chlo, *Chlorella vulgaris*; Sp1, Spirulina (*Arthrospira platensis*) obtained from University of Texas; Sp2, Spirulina (Ltd. “Spila“).

**Table 3:** Micro- and macroelement concentrations in algal extract samples.

Trace Element	Algal Extract Samples					
	Macroalgae				Microalgae	
	Cla	Furc	Ul	Chlo	Sp1	Sp2
	Macro-elements, mg kg^−1^ d.m.					
Na	220 ± 13 a,C	277 ± 11 b,D	517 ± 25 c,F	27.1 ± 1.3 a,A	136 ± 6 b,B	458 ± 18 c,E
Mg	140 ± 9 a,D	206 ± 14 b,E	259 ± 14 c,F	11.7 ± 0.9 a,A	15.5 ± 1.1 b,B	51.0 ± 3.2 c,C
K	975 ± 32 c,E	445 ± 21 b,D	336 ± 19 a,C	77 ± 4 a,A	110 ± 5 b,B	950 ± 29 c,E
Ca	40.2 ± 3.1 b,E	68.3 ± 4.2 c,F	18.8 ± 0.7 a,C	2.36 ± 0.14 a,A	7.17 ± 0.16 b,B	29.1 ± 1.7 c,D
	Essential microelements, mg kg^−1^ d.m.					
Cr	0.003 ± 0.001 a,A	0.042 ± 0.003 c,C	0.023 ± 0.002 b,B	0.002 ± 0.001 a,A	0.005 ± 0.002 a,A	0.056 ± 0.004 b,D
Mn	7.41 ± 0.52 c,F	1.10 ± 0.07 b,E	0.435 ± 0.021 a,D	0.028 ± 0.002 a,A	0.063 ± 0.004 b,B	0.333 ± 0.021 c,C
Fe	4.34 ± 0.31 c,E	2.66 ± 0.19 b,D	1.09 ± 0.08 a,A	2.25 ± 0.011 b,C	1.37 ± 0.009 a,B	4.73 ± 0.15 c,E
Co	0.064 ± 0.005 d,D	0.003 ± 0.001 a,A	0.006 ± 0.001 b,B	0.002 ± 0.001 a,A	0.002 ± 0.001 a,A	0.040 ± 0.003 c,C
Ni	0.301 ± 0.011 c,D	0.015 ± 0.002 b,B	0.005 ± 0.001 a,A	nd	nd	0.020 ± 0.001 C
Cu	0.101 ± 0.009 b,D	0.004 ± 0.001 a,A	0.202 ± 0.013 c,E	0.077 ± 0.003 b,C	0.020 ± 0.002 a,B	0.071 ± 0.005 b,C
Se	0.004 ± 0.002 a,A	0.001 ± 0.001 a,A	0.002 ± 0.001 a,A	nd	nd	0.004 ± 0.002 A
	Non-essential microelements, mg kg^−1^ d.m.					
As	0.334 ± 0.021 b,D	0.255 ± 0.016 a,C	0.437 ± 0.031 c,E	0.004 ± 0.002 a,A	0.002 ± 0.001 a,A	0.022 ± 0.002 b,B
V	0.006 ± 0.001 a,B	0.004 ± 0.001 a,A,B	0.004 ± 0.001 a,A,B	0.001 ± 0.001 a,A	nd	0.002 ± 0.001 a,A
Rb	0.507 ± 0.026 c,F	0.252 ± 0.017 b,E	0.140 ± 0.009 a,C	0.020 ± 0.001 b,B	0.010 ± 0.001 a,A	0.181 ± 0.012 c,D
Sr	0.398 ± 0.014 b,E	0.621 ± 0.035 c,F	0.245 ± 0.011 a,C	0.017 ± 0.002 a,A	0.060 ± 0.003 b,B	0.346 ± 0.019 c,D
Mo	nd	0.004 ± 0.001 a,A	0.007 ± 0.001 b,B	nd	0.004 ± 0.001 a,A	0.010 ± 0.001 b,C
Ag	0.018 ± 0.001 a,A	0.098 ± 0.007 c,C	0.029 ± 0.002 b,B	nd	0.034 ± 0.003 a,B	0.164 ± 0.013 b,D
Sb	0.046 ± 0.003 b,C	0.005 ± 0.001 a,A	0.163 ± 0.007 c,E	nd	0.032 ± 0.003 a,B	0.098 ± 0.008 b,D
Cs	nd	0.001 ± 0.000	nd	nd	nd	nd
Ti	nd	nd	nd	nd	nd	0.001 ± 0.000
Cd	nd	nd	nd	nd	nd	0.001 ± 0.000
Ba	0.032 ± 0.002 b,C	0.033 ± 0.002 b,C	0.008 ± 0.001 a,B	0.001 ± 0.000 a,A	0.006 ± 0.001 b,B	0.043 ± 0.003 c,D
Pb	0.002 ± 0.001 a,A	0.003 ± 0.001 a,b,A,B	0.001 ± 0.000 a,A	0.001 ± 0.000 a,A	0.001 ± 0.000 a,A	0.002 ± 0.001 a,A
Al	0.343 ± 0.018 b,B	1.14 ± 0.01 c,C	0.111 ± 0.009 a,A	nd	nd	1.15 ± 0.009 C
Li	0.013 ± 0.002 a,B	0.023 ± 0.002 b,C	0.047 ± 0.003 c,D	0.002 ± 0.001 a,A	0.005 ± 0.002 a,A	0.070 ± 0.006 b,E

Cla, *Cladophora rupestris*; Ul, *Ulva intestinalis*; Furc, *Furcellaria lumbricalis*; Chlo, *Chlorella vulgaris*; Sp1, Spirulina (*Arthrospira platensis*) obtained from the University of Texas; Sp2, Spirulina (Ltd. “Spila“). Data are represented as means (n = 3, replicates of analysis) ± SD. a–c for the same analytical parameters, in macro- and microalgae groups, means with different letters are significantly different (*p* ≤ 0.05). A–F for the same analytical parameters, in all algal samples, means with different letters are significantly different (*p* ≤ 0.05); nd - not determined.

## Data Availability

The data are available from the corresponding author upon reasonable request.

## Supplementary Materials

The following are available online at https://www.mdpi.com/article/10.3390/foods10092226/s1, Supplementary File S1: Raw data file.

## References

[1] Blumberga D., Chen B., Ozarska A., Indzere Z., Lauka D. (2019). Energy, Bioeconomy, Climate Changes and Environment Nexus. Environ. Clim. Technol..

[2] Pastare L., Romagnoli F. (2019). Life Cycle Cost Analysis of Biogas Production from, and in Latvian Conditions. Environ. Clim. Technol..

[3] Zihare L., Gusca J., Spalvins K., Blumberga D. (2019). Priorities Determination of Using Bioresources. Case Study of Heracleum Sosnowskyi. Sci. J. Riga Tech. Univ. Environ. Clim. Technol..

[4] Díaz-Reinoso B., Torres M.D., Kraan S., Dominguez H. (2020). Chapter 14—Concentration and purification of seaweed extracts using membrane technologies. Sustainable Seaweed Technologies.

[5] Kumar B.R., Mathimani T., Sudhakar M.P., Rajendran K., Nizami A.-S., Brindhadevi K., Pugazhendhi A. (2021). A State of the Art Review on the Cultivation of Algae for Energy and Other Valuable Products: Application, Challenges, and Opportunities. Renew. Sustain. Energy Rev..

[6] Khan M.N., Khorshid Abbas Z. (2015). Variation in Photosynthetic Pigments, Antioxidant Enzymes and Osmolyte Accumulation in Seaweeds of Red Sea. Int. J. Plant Biol. Res..

[7] Nazarudin M.F., Isha A., Mastuki S.N., Ain N.M., Mohd Ikhsan N.F., Abidin A.Z., Aliyu-Paiko M. (2020). Chemical Composition and Evaluation of the α-Glucosidase Inhibitory and Cytotoxic Properties of Marine Algae Ulva Intestinalis, Halimeda Macroloba, and Sargassum Ilicifolium. Evid. Based Complement. Alternat. Med..

[8] Michalak I., Messyasz B. (2021). Concise Review of *Cladophora* spp.: Macroalgae of Commercial Interest. J. Appl. Phycol..

[9] Khuantrairong T., Traichaiyaporn S. (2011). The Nutritional Value of Edible Freshwater Alga *Cladophora* sp. (Chlorophyta) Grown under Different Phosphorus Concentrations. Int. J. Agric. Biol. Pak..

[10] Joyce K.E., Phinn S.R. (2003). Hyperspectral Analysis of Chlorophyll Content and Photosynthetic Capacity of Coral Reef Substrates. Limnol. Oceanogr..

[11] Vega J., Álvarez-Gómez F., Güenaga L., Figueroa F.L., Gómez-Pinchetti J.L. (2020). Antioxidant Activity of Extracts from Marine Macroalgae, Wild-Collected and Cultivated, in an Integrated Multi-Trophic Aquaculture System. Aquaculture.

[12] Peñuelas J., Filella I. (1998). Visible and Near-Infrared Reflectance Techniques for Diagnosing Plant Physiological Status. Trends Plant Sci..

[13] Kirst G.O. (1990). Salinity Tolerance of Eukaryotic Marine Algae. Annu. Rev. Plant Physiol. Plant Mol. Biol..

[14] Kotta J., Möller T., Orav-Kotta H., Pärnoja M. (2014). Realized Niche Width of a Brackish Water Submerged Aquatic Vegetation under Current Environmental Conditions and Projected Influences of Climate Change. Mar. Environ. Res..

[15] Vahtmäe E., Kotta J., Orav-Kotta H., Kotta I., Pärnoja M., Kutser T. (2018). Predicting Macroalgal Pigments (Chlorophyll a, Chlorophyll b, Chlorophyll a + b, Carotenoids) in Various Environmental Conditions Using High-Resolution Hyperspectral Spectroradiometers. Int. J. Remote Sens..

[16] Yokoya N.S., Necchi O., Martins A.P., Gonzalez S.F., Plastino E.M. (2007). Growth Responses and Photosynthetic Characteristics of Wild and Phycoerythrin-Deficient Strains of Hypnea Musciformis (Rhodophyta). J. Appl. Phycol..

[17] Ji N.K., Kumar R.N., Bora A., Amb M.K., Chakraborthy S. (2009). An Evaluation of the Pigment Composition of Eighteen Marine Macroalgae Collected from Okha Coast, Gulf of Kutch, India. Our Nat..

[18] Heriyanto H., Juliadiningtyas A., Shioi Y., Limantara L., Brotosudarmo T. (2017). Analysis of Pigment Composition of Brown Seaweeds Collected from Panjang Island, Central Java, Indonesia. Philipp. J. Sci..

[19] Rinawati M., Sari L.A., Pursetyo K.T. Chlorophyll and Carotenoids Analysis Spectrophotometer Using Method on Microalgae. Proceedings of the IOP Conference Series: Earth and Environmental Science.

[20] Fernando I.P.S., Kim M., Son K.-T., Jeong Y., Jeon Y.-J. (2016). Antioxidant Activity of Marine Algal Polyphenolic Compounds: A Mechanistic Approach. J. Med. Food.

[21] Souza B.W.S., Cerqueira M.A., Bourbon A.I., Pinheiro A.C., Martins J.T., Teixeira J.A., Coimbra M.A., Vicente A.A. (2012). Chemical Characterization and Antioxidant Activity of Sulfated Polysaccharide from the Red Seaweed Gracilaria Birdiae. Food Hydrocoll..

[22] Torres P., Santos J.P., Chow F., Pena Ferreira M.J., dos Santos D.Y.A.C. (2018). Comparative Analysis of in Vitro Antioxidant Capacities of Mycosporine-like Amino Acids (MAAs). Algal Res..

[23] Amsler C.D., Fairhead V.A. (2006). Defensive and Sensory Chemical Ecology of Brown Algae.

[24] Jormalainen V., Honkanen T., Amsler C.D. (2008). Macroalgal Chemical Defenses and Their Roles in Structuring Temperate Marine Communities. Algal Chemical Ecology.

[25] Pavia H., Toth G.B. (2000). Influence of Light and Nitrogen on the Phlorotannin Content of the Brown Seaweeds Ascophyllum Nodosum and Fucus Vesiculosus. Hydrobiologia.

[26] Sobhan R., Sternberg S.P.K. (1999). Cadmium Removal Using Cladophora. J. Environ. Sci. Health Part A.

[27] Bačkorová M., Maslaňáková I., Bačkor M. (2016). Copper Uptake and Copper-Induced Physiological Changes in the Marine Alga Cladophora Prolifera (Roth.) Kütz. (Chlorophyta, Ulvophyceae). Braz. J. Bot..

[28] Ebadi A.G., Hisoriev H. (2017). The Prevalence of Heavy Metals in *Cladophora glomerata* L. from Farahabad Region of Caspian Sea—Iran. Toxicol. Environ. Chem..

[29] Zbikowski R., Szefer P., Latała A. (2007). Comparison of Green Algae *Cladophora* sp. and *Enteromorpha* sp. as Potential Biomonitors of Chemical Elements in the Southern Baltic. Sci. Total Environ..

[30] Akın H.K., Ünlü E. (2013). Cadmium Accumulation by Green Algae *Cladophora glomerata* (L.) Kutz. (Chlorophyta) in Presence of Nile Tilapia *Oreochromis niloticus* (L.). Toxicol. Environ. Chem..

[31] Tolpeznikaite E., Ruzauskas M., Pilkaityte R., Bartkevics V., Zavistanaviciute P., Starkute V., Lele V., Zokaityte E., Mozuriene E., Ruibys R. (2021). Influence of Fermentation on the Characteristics of Baltic Sea Macroalgae, Including Microbial Profile and Trace Element Content. Food Control.

[32] Horincar V., Parfene G., Tyagi A., Gottardi D., Dinică R., Guerzoni M.E., Bahrim G. (2013). Extraction and Characterization of Volatile Compounds and Fatty Acids from Red and Green Macroalgae from the Romanian Black Sea in Order to Obtain Valuable Bioadditives and Biopreservatives. J. Appl. Phycol..

[33] Lezcano V., Fernández C., Parodi E.R., Morelli S. (2018). Antitumor and Antioxidant Activity of the Freshwater Macroalga Cladophora Surera. J. Appl. Phycol..

[34] Munir M., Qureshi R., Bibi M., Khan A.M. (2019). Pharmaceutical Aptitude of Cladophora: A Comprehensive Review. Algal Res..

[35] Soltani S., Ebrahimzadeh M.A., Khoshrooei R., Rahmani Z. (2012). Antibacterial and Antihemolytic Activities of Enteromorpha Intestinalis in Caspian Sea Coast, Iran. J. Med. Plants Res..

[36] Srimaroeng C., Ontawong A., Saowakon N., Vivithanaporn P., Pongchaidecha A., Amornlerdpison D., Soodvilai S., Chatsudthipong V. (2015). Antidiabetic and Renoprotective Effects of Cladophora Glomerata Kützing Extract in Experimental Type 2 Diabetic Rats: A Potential Nutraceutical Product for Diabetic Nephropathy. J. Diabetes Res..

[37] Zbakh H., Chiheb I., Motilva V., Riadi H. (2014). Antibacterial, Cytotoxic and Antioxidant Potentials of Cladophora prolifera (Roth) Kutzing Collected from the Mediterranean Coast of Morocco. Am. J. Phytomedicine Clin. Ther..

[38] Torres-Tiji Y., Fields F.J., Mayfield S.P. (2020). Microalgae as a Future Food Source. Biotechnol. Adv..

[39] Dere S., Güneş T., Sivaci R. (1998). Spectrophotometric Determination of Chlorophyll-A, B and Total Carotenoid Contents of Some Algae Species Using Different Solvents. Turk. J. Bot..

[40] Sakalauskaitė J., Viskelis P., Dambrauskienė E., Sakalauskienė S., Samuolienė G., Brazaitytė A., Duchovskis P., Urbonavičienė D. (2013). The Effects of Different UV-B Radiation Intensities on Morphological and Biochemical Characteristics in *Ocimum basilicum* L.. J. Sci. Food Agric..

[41] Nayek S., Haque C.I., Nishika J., Roy S. (2014). Spectrophotometric Analysis of Chlorophylls and Carotenoids from Commonly Grown Fern Species by Using Various Extracting Solvents. Res. J. Chem. Sci..

[42] Ainsworth E.A., Gillespie K.M. (2007). Estimation of Total Phenolic Content and Other Oxidation Substrates in Plant Tissues Using Folin-Ciocalteu Reagent. Nat. Protoc..

[43] Brand-Williams W., Cuvelier M.E., Berset C. (1995). Use of a Free Radical Method to Evaluate Antioxidant Activity. LWT Food Sci. Technol..

[44] Urbonaviciene D., Viskelis P., Viškelis J., Jankauskiene J., Bobinas C. (2012). Lycopene and β-Carotene in Non-Blanched and Blanched Tomatoes. J. Food Agric. Environ..

[45] Benzie I.F., Strain J.J. (1996). The Ferric Reducing Ability of Plasma (FRAP) as a Measure of “Antioxidant Power”: The FRAP Assay. Anal. Biochem..

[46] Bobinaitė R., Pataro G., Lamanauskas N., Šatkauskas S., Viškelis P., Ferrari G. (2015). Application of Pulsed Electric Field in the Production of Juice and Extraction of Bioactive Compounds from Blueberry Fruits and Their By-Products. J. Food Sci. Technol..

[47] Bartkiene E., Bartkevics V., Starkute V., Krungleviciute V., Cizeikiene D., Zadeike D., Juodeikiene G., Maknickiene Z. (2016). Chemical Composition and Nutritional Value of Seeds of *Lupinus luteus* L., *L. angustifolius* L. and New Hybrid Lines of *L. angustifolius* L.. Zemdirb. Agric..

[48] Gille A., Trautmann A., Posten C., Briviba K. (2016). Bioaccessibility of Carotenoids from Chlorella Vulgaris and Chlamydomonas Reinhardtii. Int. J. Food Sci. Nutr..

[49] Cha K.H., Lee H.J., Koo S.Y., Song D.-G., Lee D.-U., Pan C.-H. (2010). Optimization of Pressurized Liquid Extraction of Carotenoids and Chlorophylls from Chlorella Vulgaris. J. Agric. Food Chem..

[50] Kitada K., Machmudah S., Sasaki M., Goto M., Nakashima Y., Kumamoto S., Hasegawa T. (2009). Supercritical CO_2_ Extraction of Pigment Components with Pharmaceutical Importance from Chlorella Vulgaris. J. Chem. Technol. Biotechnol..

[51] Zhang H., Huang D., Cramer W.A. (1999). Stoichiometrically Bound Beta-Carotene in the Cytochrome B6f Complex of Oxygenic Photosynthesis Protects against Oxygen Damage. J. Biol. Chem..

[52] Tavanandi H.A., Vanjari P., Raghavarao K.S.M.S. (2019). Synergistic Method for Extraction of High Purity Allophycocyanin from Dry Biomass of Arthrospira Platensis and Utilization of Spent Biomass for Recovery of Carotenoids. Sep. Purif. Technol..

[53] Chuyen H.V., Eun J.-B. (2017). Marine Carotenoids: Bioactivities and Potential Benefits to Human Health. Crit. Rev. Food Sci. Nutr..

[54] Zhang J., Sun Z., Sun P., Chen T., Chen F. (2014). Microalgal Carotenoids: Beneficial Effects and Potential in Human Health. Food Funct..

[55] Tavanandi H.A., Raghavarao K.S.M.S. (2019). Recovery of Chlorophylls from Spent Biomass of Arthrospira Platensis Obtained after Extraction of Phycobiliproteins. Bioresour. Technol..

[56] Christwardana M., Nur M.M.A., Hadiyanto H. (2013). Spirulina Platensis: Potensinya sebagai bahan pangan fungsional. J. Apl. Teknol. Pangan.

[57] Hagerthey S.E., Louda J.W., Mongkronsri P. (2006). Evaluation of pigment extraction methods and a recommended protocol for periphyton chlorophyll a determination and chemotaxonomic assessment1. J. Phycol..

[58] European Food Safety Authority (2015). Scientific Opinion on the Re-Evaluation of Chlorophylls (E 140(i)) as Food Additives. EFSA J..

[59] Yilmaz C., Gökmen V. (2016). Chlorophyll. Encyclopedia of Food and Health.

[60] Faulks R., Southon S. (1997). Dietary Carotenoids. Nutr. Food Sci..

[61] Urbonavičienė D., Bobinas Č., Bobinaitė R., Raudonė L., Trumbeckaitė S., Viškelis J., Viškelis P. (2021). Composition and Antioxidant Activity, Supercritical Carbon Dioxide Extraction Extracts, and Residue after Extraction of Biologically Active Compounds from Freeze-Dried Tomato Matrix. Processes.

[62] Meléndez-Martínez A.J., Mandić A.I., Bantis F., Böhm V., Borge G.I.A., Brnčić M., Bysted A., Cano M.P., Dias M.G., Elgersma A. (2021). A Comprehensive Review on Carotenoids in Foods and Feeds: Status Quo, Applications, Patents, and Research Needs. Crit. Rev. Food Sci. Nutr..

[63] Bogacz-Radomska L., Harasym J., Piwowar A., Galanakis C.M. (2020). 10—Commercialization aspects of carotenoids. Carotenoids: Properties, Processing and Applications.

[64] Shahidi F., Zhong Y. (2010). Novel Antioxidants in Food Quality Preservation and Health Promotion. Eur. J. Lipid Sci. Technol..

[65] Shahidi F., Ambigaipalan P. (2015). Phenolics and Polyphenolics in Foods, Beverages and Spices: Antioxidant Activity and Health Effects—A Review. J. Funct. Foods.

[66] Santoso J., Yoshie Y., Suzuki T., Sakaguchi M. (2004). Polyphenolic compounds from seaweeds: Distribution and their antioxidative effect. Developments in Food Science.

[67] Wang T., Jónsdóttir R., Ólafsdóttir G. (2009). Total Phenolic Compounds, Radical Scavenging and Metal Chelation of Extracts from Icelandic Seaweeds. Food Chem..

[68] Messyasz B., Pikosz M., Treska E., Chojnacka K., Wieczorek P.P., Schroeder G., Michalak I. (2018). Biology of Freshwater Macroalgae and Their Distribution. Algae Biomass: Characteristics and Applications: Towards Algae-Based Products.

[69] Karan T., Erenler R. (2018). Fatty Acid Constituents and Anticancer Activity of Cladophora Fracta (OF Müller Ex Vahl) Kützing. Trop. J. Pharm. Res..

[70] Zarei Jeliani Z., Mashjoor S., Soleimani S., Pirian K., Sedaghat F., Yosefzadi M. (2018). Antioxidant Activity and Cytotoxicity of Organic Extracts from Three Species of Green Macroalgae of Ulvaceae from Persian Gulf. Modares J. Biotechnol..

[71] Srikong W., Bovornreungroj N., Mittraparparthorn P., Bovornreungroj P. (2017). Antibacterial and Antioxidant Activities of Differential Solvent Extractions from the Green Seaweed Ulva Intestinalis. ScienceAsia.

[72] Farasat M., Khavari-Nejad R.-A., Nabavi S.M.B., Namjooyan F. (2014). Antioxidant Activity, Total Phenolics and Flavonoid Contents of Some Edible Green Seaweeds from Northern Coasts of the Persian Gulf. Iran. J. Pharm. Res..

[73] Tepe B., Daferera D., Sokmen A., Sokmen M., Polissiou M. (2005). Antimicrobial and Antioxidant Activities of the Essential Oil and Various Extracts of Salvia Tomentosa Miller (Lamiaceae). Food Chem..

[74] Naczk M., Shahidi F. (2006). Phenolics in Cereals, Fruits and Vegetables: Occurrence, Extraction and Analysis. J. Pharm. Biomed. Anal..

[75] Naseri A., Holdt S.L., Jacobsen C. (2019). Biochemical and Nutritional Composition of Industrial Red Seaweed Used in Carrageenan Production. J. Aquat. Food Prod. Technol..

[76] Sabeena Farvin K.H., Jacobsen C. (2013). Phenolic Compounds and Antioxidant Activities of Selected Species of Seaweeds from Danish Coast. Food Chem..

[77] Tibbetts S.M., Milley J.E., Lall S.P. (2016). Nutritional Quality of Some Wild and Cultivated Seaweeds: Nutrient Composition, Total Phenolic Content and in Vitro Digestibility. J. Appl. Phycol..

[78] Taghavi Takyar M.B., Haghighat Khajavi S., Safari R. (2019). Evaluation of Antioxidant Properties of Chlorella Vulgaris and Spirulina Platensis and Their Application in Order to Extend the Shelf Life of Rainbow Trout (Oncorhynchus Mykiss) Fillets during Refrigerated Storage. LWT.

[79] Agregán R., Munekata P.E.S., Franco D., Carballo J., Barba F.J., Lorenzo J.M. (2018). Antioxidant Potential of Extracts Obtained from Macro- (Ascophyllum Nodosum, Fucus Vesiculosus and Bifurcaria Bifurcata) and Micro-Algae (Chlorella Vulgaris and Spirulina Platensis) Assisted by Ultrasound. Medicines.

[80] Chakraborty K., Lipton A.P., Paul Raj R., Vijayan K.K. (2010). Antibacterial Labdane Diterpenoids of Ulva Fasciata Delile from Southwestern Coast of the Indian Peninsula. Food Chem..

[81] Schweder T., Lindequist U., Lalk M., Ulber R., Le Gal Y. (2005). Screening for New Metabolites from Marine Microorganisms. Marine Biotechnology I..

[82] Newman D.J., Cragg G.M., Snader K.M. (2003). Natural Products as Sources of New Drugs over the Period 1981-2002. J. Nat. Prod..

[83] Dussault D., Vu K.D., Vansach T., Horgen F.D., Lacroix M. (2016). Antimicrobial Effects of Marine Algal Extracts and Cyanobacterial Pure Compounds against Five Foodborne Pathogens. Food Chem..

[84] Kandhasamy M., Arunachalam K.D. (2008). Evaluation of in Vitro Antibacterial Property of Seaweeds of Southeast Coast of India. Afr. J. Biotechnol..

[85] Pesando D., Caram B. (1984). Screening of Marine Algae from the French Mediterranean Coast for Antibacterial and Antifungal Activity. Botanica Marina..

[86] Priyadharshini S., Bragadeeswaran S., Prabhu K., Ran S.S. (2011). Antimicrobial and Hemolytic Activity of Seaweed Extracts Ulva Fasciata (Delile 1813) from Mandapam, Southeast Coast of India. Asian Pac. J. Trop. Biomed..

[87] Ibtissam C., Hassane R., José M.-L., Seglar D., Francisco J., Vidal G., Antonio J., Bouziane H., Kadiri M. (2010). Screening of Antibacterial Activity in Marine Green and Brown Macroalgae from the Coast of Morocco. Afr. J. Biotechnol..

[88] Lustigman B., Brown C. (1991). Antibiotic Production by Marine Algae Isolated from the New York/New Jersey Coast. Bull. Environ. Contam. Toxicol..

[89] Manilal A., Sujith S., Sabarathnam B., Kiran G.S., Selvin J., Shakir C., Lipton A.P. (2010). Bioactivity of the Red Algae Asparagopsis Taxiformis Collected from the Southwestern Coast of India. Braz. J. Oceanogr..

[90] Kumar K.A., Rengasamy R. (2000). Evaluation of Antibacterial Potential of Seaweeds Occurring along the Coast of Tamil Nadu, India against the Plant Pathogenic Bacterium Xanthomonas Oryzae Pv. Oryzae (Ishiyama) Dye. J. Coast. Life Med..

[91] Patra A.K., Acharya B.C., Mohapatra A. (2009). Occurrence and Distribution of Bacterial Indicators and Pathogens in Coastal Waters of Orissa. IJMS.

[92] Salvador N., Garreta A.G., Lavelli L., Ribera M.A. (2007). Antimicrobial Activity of Iberian Macroalgae. Sci. Mar..

[93] Vallinayagam K., Arumugam R., Kannan R., Thirumaran G., Anantharaman P. (2009). Antibacterial Activity of Seaweeds of Pudumadam Coast. Glob. J. Pharmacol..

[94] Bennett P.M. (2008). Plasmid Encoded Antibiotic Resistance: Acquisition and Transfer of Antibiotic Resistance Genes in Bacteria. Br. J. Pharmacol..

[95] Fischbach M.A., Walsh C.T. (2009). Antibiotics for Emerging Pathogens. Science.

[96] Kiran N., Siddiqui G., Khan A.N., Khan I., Pavase T. (2014). Extraction and Screening of Bioactive Compounds with Antimicrobial Properties from Selected Species of Mollusk and Crustacean. J. Clin. Cell Immunol..

[97] Lee K.-A., Moon S.H., Kim K.-T., Mendonca A.F., Paik H.-D. (2010). Antimicrobial Effects of Various Flavonoids on *Escherichia coli* O157:H7 Cell Growth and Lipopolysaccharide Production. Food Sci. Biotechnol..

[98] Li X.-Z., Nikaido H. (2009). Efflux-Mediated Drug Resistance in Bacteria. Drugs.

[99] Stix G. (2006). An Antibiotic Resistance Fighter. Sci. Am..

[100] Wright G.D., Sutherland A.D. (2007). New Strategies for Combating Multidrug-Resistant Bacteria. Trends Mol. Med..

[101] Rizwan S., Siddiqui G., Shoaib M., Mahmood K., Ul-Hassan H. (2020). Antibacterial Activity of Ulva Intestinalis, U. Faciata, and U. Lactuca against Biofilm-Associated Bacteria. Egypt. J. Aquat. Biol. Fish..

[102] Shoaib M., Burhan Z.-N., Shafique S., Jabeen H., Jamal P., Siddique A. (2017). Phytoplankton Composition in a Mangrove Ecosystem at Sandspit, Karachi, Pakistan. Pak. J. Bot.

[103] Hellio C., Bremer G., Pons A.M., Le Gal Y., Bourgougnon N. (2000). Inhibition of the Development of Microorganisms (Bacteria and Fungi) by Extracts of Marine Algae from Brittany, France. Appl. Microbiol. Biotechnol..

[104] Lima-Filho J.V.M., Carvalho A.F.F.U., Freitas S.M., Melo V.M.M. (2002). Antibacterial Activity of Extracts of Six Macroalgae from the Northeastern Brazilian Coast. Braz. J. Microbiol..

[105] Moreau J., Pesando D., Bernard P., Caram B., Pionnat J.C. (1988). Seasonal Variations in the Production of Antifungal Substances by Some Dictyotales (Brown Algae) from the French Mediterranean Coast. Hydrobiologia.

[106] Osman M.E.H., Abushady A.M., Elshobary M.E. (2010). In Vitro Screening of Antimicrobial Activity of Extracts of Some Macroalgae Collected from Abu-Qir Bay Alexandria, Egypt. Afr. J. Biotechnol..

[107] Perez G.R.M., Avila A.J.G., Perez G.S., Martinez C.A., Martinez C.G. (1990). Antimicrobial Activity of Some American Algae. J. Ethnopharmacol..

[108] Val A., Platas G., Basilio A., Cabello A., Gorrochategui J., Suay I., Vicente F., Portillo E., Río M., Reina G. (2001). Screening of Antimicrobial Activities in Red, Green and Brown Macroalgae from Gran Canaria (Canary Islands, Spain). Int. Microbiol..

[109] Burkholder P., Burkholder L., Almodovar L. (1960). Antibiotic Activity of Some Marine Algae of Puerto Rico. Bot. Mar..

[110] Newton L. (1956). The Production of Antibiotic Substances by Seaweeds. Phycol. Bull..

[111] Kumar S.R., Ramanathan G., Subhakaran M., Inbaneson S.J. (2009). Antimicrobial Compounds from Marine Halophytes for Silkworm Disease Treatment. Int. J. Med. Med. Sci..

[112] Desbois A.P., Smith V.J. (2010). Antibacterial Free Fatty Acids: Activities, Mechanisms of Action and Biotechnological Potential. Appl. Microbiol. Biotechnol..

[113] Gupta S., Abu-Ghannam N. (2011). Recent Developments in the Application of Seaweeds or Seaweed Extracts as a Means for Enhancing the Safety and Quality Attributes of Foods. Innov. Food Sci. Emerg. Technol..

[114] Kamenarska Z., Stefanov K., Dimitrova-Konaklieva S., Najdenski H., Tsvetkova I., Popov S. (2004). Chemical Composition and Biological Activity of the Brackish-Water Green Alga *Cladophora rivularis* (L.) Hoek. Botanica Marina..

[115] Laungsuwon R., Chulalaksananukul W. (2014). Chemical Composition and Antibacterial Activity of Extracts from Freshwater Green Algae, Cladophora Glomerata Kützing and Microspora Floccosa (Vaucher) Thuret. J. Biosci. Biotechnol..

[116] Stabili L., Acquaviva M.I., Biandolino F., Cavallo R.A., De Pascali S.A., Fanizzi F.P., Narracci M., Cecere E., Petrocelli A. (2014). Biotechnological Potential of the Seaweed Cladophora Rupestris (Chlorophyta, Cladophorales) Lipidic Extract. New Biotechnol..

[117] Alshuniaber M.A., Krishnamoorthy R., AlQhtani W.H. (2021). Antimicrobial Activity of Polyphenolic Compounds from Spirulina against Food-Borne Bacterial Pathogens. Saudi J. Biol. Sci..

[118] Piette A., Verschraegen G. (2009). Role of Coagulase-Negative Staphylococci in Human Disease. Vet. Microbiol..

[119] Barros E.M., Ceotto H., Bastos M.C.F., dos Santos K.R.N., Giambiagi-deMarval M. (2012). Staphylococcus Haemolyticus as an Important Hospital Pathogen and Carrier of Methicillin Resistance Genes. J. Clin. Microbiol..

[120] Chiew Y.-F., Charles M., Johnstone M.C., Thompson K.M., Parnell K.D., Penno E.C. (2007). Detection of Vancomycin Heteroresistant Staphylococcus Haemolyticus and Vancomycin Intermediate Resistant Staphylococcus Epidermidis by Means of Vancomycin Screening Agar. Pathology.

[121] Moura G.S., Mota R.A., Marques M.F.S., Abad A.C.A., Costa L.B.B.C., Souza F.N., Almeida V.M., Filho G.B.S., Bom H.A.S.C., Klaumann F. (2021). Gangrenous Mastitis in Sheep Caused by Multidrug-Resistant *Staphylococcus Haemolyticus*. Pesqui. Veterinária Bras..

[122] Menteş Ö., Ercan R., Akçelik M. (2007). Inhibitor Activities of Two Lactobacillus Strains, Isolated from Sourdough, against Rope-Forming Bacillus Strains. Food Control.

[123] Sandulachi E., Bulgaru V., Ghendov-Mosanu A., Sturza R. (2021). Controlling the Risk of Bacillus in Food Using Berries. Food Nutr. Sci..

[124] Han K., Jung E.-G., Kwon H.-J., Patnaik B.B., Baliarsingh S., Kim W.-J., Nam K.-W., Lee J.-S., Han M.-D., Kang S.W. (2021). Gene Expression Analysis of Inflammation-Related Genes in Macrophages Treated with α-(1 → 3, 1 → 6)-D-Glucan Extracted from Streptococcus Mutans. Int. J. Biol. Macromol..

[125] Jang H.J., Kim J.H., Lee N.-K., Paik H.-D. (2021). Inhibitory Effects of Lactobacillus Brevis KU15153 against Streptococcus Mutans KCTC 5316 Causing Dental Caries. Microb. Pathog..

[126] Yue J., Yang H., Liu S., Song F., Guo J., Huang C. (2018). Influence of Naringenin on the Biofilm Formation of Streptococcus Mutans. J. Dent..

[127] Lim S.-M., Lee N.-K., Kim K.-T., Paik H.-D. (2020). Probiotic Lactobacillus Fermentum KU200060 Isolated from Watery Kimchi and Its Application in Probiotic Yogurt for Oral Health. Microb. Pathog..

[128] Balasubramanian A.R., Vasudevan S., Shanmugam K., Lévesque C.M., Solomon A.P., Neelakantan P. (2021). Combinatorial Effects of Trans-Cinnamaldehyde with Fluoride and Chlorhexidine on Streptococcus Mutans. J. Appl. Microbiol..

[129] Ajaegbu E.E., Ezeh C.U., Dieke A.J., Onuora A.L., Ugochukwu J.I. (2020). Antimicrobial Efficacy of Toothpastes Containing Fluoride against Clinically Isolated Streptococci Mutans. Adv. Res..

[130] Lim S.-M., Lee N.-K., Paik H.-D. (2020). Antibacterial and Anticavity Activity of Probiotic Lactobacillus Plantarum 200661 Isolated from Fermented Foods against Streptococcus Mutans. LWT.

[131] Nakamura E., Yokota H., Matsui T. (2012). The in Vitro Digestibility and Absorption of Magnesium in Some Edible Seaweeds. J. Sci. Food Agric..

[132] Agency I.A.E., World Health Organization (1996). Nations, Food and Agriculture Organization of the U. Trace Elements in Human Nutrition and Health.

[133] Waheed M., Butt M.S., Shehzad A., Adzahan N.M., Shabbir M.A., Rasul Suleria H.A., Aadil R.M. (2019). Eggshell Calcium: A Cheap Alternative to Expensive Supplements. Trends Food Sci. Technol..

[134] Rzymski P., Budzulak J., Niedzielski P., Klimaszyk P., Proch J., Kozak L., Poniedziałek B. (2019). Essential and Toxic Elements in Commercial Microalgal Food Supplements. J. Appl. Phycol..

[135] Thompson M.E.H., Raizada M.N. (2018). Fungal Pathogens of Maize Gaining Free Passage Along the Silk Road. Pathogens.

[136] (2013). Scientific Opinion on Dietary Reference Values for Manganese. EFSA J..

[137] (2009). EFSA Scientific Opinion on the Use of Cobalt Compounds as Additives in Animal Nutrition. EFSA J..

[138] World Health Organization, Regional Office for Europe (2000). Air Quality Guidelines for Europe.

[139] García-Casal M.N., Pereira A.C., Leets I., Ramírez J., Quiroga M.F. (2007). High Iron Content and Bioavailability in Humans from Four Species of Marine Algae. J. Nutr..

[140] European Food Safety Authority, European Food Safety Authority (2006). Tolerable Upper Intake Levels for Vitamins and Minerals.

[141] Institute of Medicine (US) Panel on Micronutrients (2001). Dietary Reference Intakes for Vitamin A, Vitamin K, Arsenic, Boron, Chromium, Copper, Iodine, Iron, Manganese, Molybdenum, Nickel, Silicon, Vanadium, and Zinc.

[142] Desideri D., Cantaluppi C., Ceccotto F., Meli M.A., Roselli C., Feduzi L. (2016). Essential and Toxic Elements in Seaweeds for Human Consumption. J. Toxicol. Environ. Health A.

[143] Burger J., Gochfeld M., Jeitner C., Donio M., Pittfield T. (2012). Lead (Pb) in Biota and Perceptions of Pb Exposure at a Recently Designated Superfund Beach Site in New Jersey. J. Toxicol. Environ. Health A.

[144] Hwang Y.O., Park S.G., Park G.Y., Choi S.M., Kim M.Y. (2010). Total Arsenic, Mercury, Lead, and Cadmium Contents in Edible Dried Seaweed in Korea. Food Addit. Contam. Part B Surveill..

[145] Feldmann J., Krupp E.M. (2011). Critical Review or Scientific Opinion Paper: Arsenosugars—A Class of Benign Arsenic Species or Justification for Developing Partly Speciated Arsenic Fractionation in Foodstuffs?. Anal. Bioanal. Chem..

[146] Commission Regulations Commission Regulation (EC) No 1881/2006 of 19 December 2006 Setting Maximum Levels for Certain Contaminants in Foodstuffs (Text with EEA Relevance). https://www.legislation.gov.uk/eur/2006/1881.

[147] Anna F., Lubecki L., Szymczak-Żyła M., Kowalewska G., Radosław Ż., Piotr S. (2008). Utilisation of Macroalgae from the Sopot Beach (Baltic Sea). Oceanologia.

[148] Commission Directive 2002/32/EC EUR-Lex—32002L0032—EN—EUR-Lex. https://eur-lex.europa.eu/legal-content/EN/ALL/?uri=CELEX%3A32002L0032.

[149] Papazi A., Makridis P., Divanach P. (2010). Harvesting Chlorella Minutissima Using Cell Coagulants. J. Appl. Phycol..

[150] Rzymski P., Niedzielski P., Kaczmarek N., Jurczak T., Klimaszyk P. (2015). The Multidisciplinary Approach to Safety and Toxicity Assessment of Microalgae-Based Food Supplements Following Clinical Cases of Poisoning. Harmful Algae.

