# Expression of Concern: Tolpeznikaite et al. Characterization of Macro- and Microalgae Extracts Bioactive Compounds and Micro- and Macroelements Transition from Algae to Extract. *Foods* 2021, *10*, 2226

**DOI:** 10.3390/foods13142249

**Published:** 2024-07-17

**Authors:** Foods Editorial Office

**Affiliations:** MDPI, St. Alban-Anlage 66, 4052 Basel, Switzerland; foods@mdpi.com

With this notice, the *Foods* Editorial Office alerts the readers to concerns related to this article [1]. Following publication, concerns were raised to the Editorial Office regarding the integrity of the data presented in this study. The Editor-in-Chief has decided to issue this expression of concern while an investigation is being conducted. The authors’ institution has been contacted to contribute to this investigation.

Once this process has been completed, the Editorial Office will provide an update on this situation and announce any post-publication modification deemed to be necessary, as per MDPI’s Update to Published Papers policy.
