# Correction: Tolpeznikaite et al. Characterization of Macro- and Microalgae Extracts Bioactive Compounds and Micro- and Macroelements Transition from Algae to Extract. *Foods* 2021, *10*, 2226

**DOI:** 10.3390/foods14091539

**Published:** 2025-04-28

**Authors:** Ernesta Tolpeznikaite, Vadims Bartkevics, Modestas Ruzauskas, Renata Pilkaityte, Pranas Viskelis, Dalia Urbonaviciene, Paulina Zavistanaviciute, Egle Zokaityte, Romas Ruibys, Elena Bartkiene

**Affiliations:** 1 Faculty of Animal Sciences, Institute of Animal Rearing Technologies, Lithuanian University of Health Sciences, Mickeviciaus Str. 9, LT-44307 Kaunas, Lithuania; ernesta.tolpeznikaite@lsmuni.lt (E.T.); paulina.zavistanaviciute@lsmuni.lt (P.Z.); egle.zokaityte@lsmuni.lt (E.Z.); 2 Institute of Food Safety, Animal Health and Environment “BIOR”, Lejupes iela 3, Zemgales priekšpilsēta, LV-1076 Riga, Latvia; vadims.bartkevics@bior.gov.lv; 3 Department of Anatomy and Physiology, Faculty of Veterinary, Lithuanian University of Health Sciences, Mickeviciaus Str. 9, LT-44307 Kaunas, Lithuania; modestas.ruzauskas@lsmuni.lt; 4 Faculty of Veterinary, Institute of Microbiology and Virology, Lithuanian University of Health Sciences, Mickeviciaus Str. 9, LT-44307 Kaunas, Lithuania; 5 Marine Research Institute, Klaipėda University, Universiteto Ave. 17, LT-92294 Klaipėda, Lithuania; renata.pilkaityte@apc.ku.lt; 6 Lithuanian Research Centre for Agriculture and Forestry, Institute of Horticulture, Kauno Str. 30, LT-54333 Babtai, Lithuania; pranas.viskelis@lammc.lt (P.V.); dalia.urbonaviciene@lammc.lt (D.U.); 7 Department of Food Safety and Quality, Faculty of Veterinary, Lithuanian University of Health Sciences, Mickeviciaus Str. 9, LT-44307 Kaunas, Lithuania; 8 Institute of Agricultural and Food Sciences, Agriculture Academy, Vytautas Magnus University, K. Donelaicio Str. 58, LT-44244 Kaunas, Lithuania; romas.ruibys@vdu.lt

In the original publication [1], changes are needed for Figure 2, because of a technical mistake in the legend. There were two identical designations for different parameters (Chlorophyll a; Chlorophyl a). This should be corrected to Chlorophyl a; Chlorophyl b. The corrected Figure 2 is provided below.

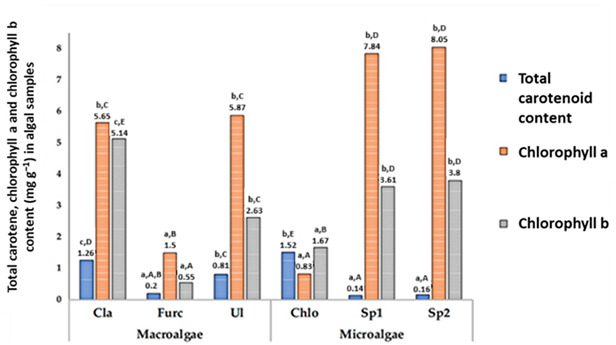



In Table 3, because of a technical mistake, incorrect units were included (g kg^−1^ should be changed to mg kg^−1^). Because of the latter changes, corrections in Section “3. Results and Discussion” subsection “3.4. Micro- and Macroelement Concentrations in Algal Samples” should be performed. The corrected subsection “3.4. Micro- and Macroelement Concentrations in Algal Samples” is provided after Table 3.

*3.4. Micro- and Macroelement Concentrations in Algal Samples*

Due to a technical mistake, the concentration units were changed throughout Section 3.4 from g kg^−1^ to mg kg^−1^.

The authors state that the scientific conclusions are unaffected. This correction was approved by the Academic Editor. The original publication has also been updated.

## Figures and Tables

**Table 3:** Micro- and macroelement concentrations in algal extract samples.

Trace Element	Algal Extract Samples					
	Macroalgae				Microalgae	
	Cla	Furc	Ul	Chlo	Sp1	Sp2
	Macro-elements, mg kg^−1^ d.m.					
Na	220 ± 13 a,C	277 ± 11 b,D	517 ± 25 c,F	27.1 ± 1.3 a,A	136 ± 6 b,B	458 ± 18 c,E
Mg	140 ± 9 a,D	206 ± 14 b,E	259 ± 14 c,F	11.7 ± 0.9 a,A	15.5 ± 1.1 b,B	51.0 ± 3.2 c,C
K	975 ± 32 c,E	445 ± 21 b,D	336 ± 19 a,C	77 ± 4 a,A	110 ± 5 b,B	950 ± 29 c,E
Ca	40.2 ± 3.1 b,E	68.3 ± 4.2 c,F	18.8 ± 0.7 a,C	2.36 ± 0.14 a,A	7.17 ± 0.16 b,B	29.1 ± 1.7 c,D
	Essential microelements, mg kg^−1^ d.m.					
Cr	0.003 ± 0.001 a,A	0.042 ± 0.003 c,C	0.023 ± 0.002 b,B	0.002 ± 0.001 a,A	0.005 ± 0.002 a,A	0.056 ± 0.004 b,D
Mn	7.41 ± 0.52 c,F	1.10 ± 0.07 b,E	0.435 ± 0.021 a,D	0.028 ± 0.002 a,A	0.063 ± 0.004 b,B	0.333 ± 0.021 c,C
Fe	4.34 ± 0.31 c,E	2.66 ± 0.19 b,D	1.09 ± 0.08 a,A	2.25 ± 0.011 b,C	1.37 ± 0.009 a,B	4.73 ± 0.15 c,E
Co	0.064 ± 0.005 d,D	0.003 ± 0.001 a,A	0.006 ± 0.001 b,B	0.002 ± 0.001 a,A	0.002 ± 0.001 a,A	0.040 ± 0.003 c,C
Ni	0.301 ± 0.011 c,D	0.015 ± 0.002 b,B	0.005 ± 0.001 a,A	nd	nd	0.020 ± 0.001 C
Cu	0.101 ± 0.009 b,D	0.004 ± 0.001 a,A	0.202 ± 0.013 c,E	0.077 ± 0.003 b,C	0.020 ± 0.002 a,B	0.071 ± 0.005 b,C
Se	0.004 ± 0.002 a,A	0.001 ± 0.001 a,A	0.002 ± 0.001 a,A	nd	nd	0.004 ± 0.002 A
	Non-essential microelements, mg kg^−1^ d.m.					
As	0.334 ± 0.021 b,D	0.255 ± 0.016 a,C	0.437 ± 0.031 c,E	0.004 ± 0.002 a,A	0.002 ± 0.001 a,A	0.022 ± 0.002 b,B
V	0.006 ± 0.001 a,B	0.004 ± 0.001 a,A,B	0.004 ± 0.001 a,A,B	0.001 ± 0.001 a,A	nd	0.002 ± 0.001 a,A
Rb	0.507 ± 0.026 c,F	0.252 ± 0.017 b,E	0.140 ± 0.009 a,C	0.020 ± 0.001 b,B	0.010 ± 0.001 a,A	0.181 ± 0.012 c,D
Sr	0.398 ± 0.014 b,E	0.621 ± 0.035 c,F	0.245 ± 0.011 a,C	0.017 ± 0.002 a,A	0.060 ± 0.003 b,B	0.346 ± 0.019 c,D
Mo	nd	0.004 ± 0.001 a,A	0.007 ± 0.001 b,B	nd	0.004 ± 0.001 a,A	0.010 ± 0.001 b,C
Ag	0.018 ± 0.001 a,A	0.098 ± 0.007 c,C	0.029 ± 0.002 b,B	nd	0.034 ± 0.003 a,B	0.164 ± 0.013 b,D
Sb	0.046 ± 0.003 b,C	0.005 ± 0.001 a,A	0.163 ± 0.007 c,E	nd	0.032 ± 0.003 a,B	0.098 ± 0.008 b,D
Cs	nd	0.001 ± 0.000	nd	nd	nd	nd
Ti	nd	nd	nd	nd	nd	0.001 ± 0.000
Cd	nd	nd	nd	nd	nd	0.001 ± 0.000
Ba	0.032 ± 0.002 b,C	0.033 ± 0.002 b,C	0.008 ± 0.001 a,B	0.001 ± 0.000 a,A	0.006 ± 0.001 b,B	0.043 ± 0.003 c,D
Pb	0.002 ± 0.001 a,A	0.003 ± 0.001 a,b,A,B	0.001 ± 0.000 a,A	0.001 ± 0.000 a,A	0.001 ± 0.000 a,A	0.002 ± 0.001 a,A
Al	0.343 ± 0.018 b,B	1.14 ± 0.01 c,C	0.111 ± 0.009 a,A	nd	nd	1.15 ± 0.009 C
Li	0.013 ± 0.002 a,B	0.023 ± 0.002 b,C	0.047 ± 0.003 c,D	0.002 ± 0.001 a,A	0.005 ± 0.002 a,A	0.070 ± 0.006 b,E

Cla, *Cladophora rupestris*; Ul, *Ulva intestinalis*; Furc, *Furcellaria lumbricalis*; Chlo, *Chlorella vulgaris*; Sp1, Spirulina (*Arthrospira platensis*) obtained from the University of Texas; Sp2, Spirulina (Ltd. “Spila“). Data are represented as means (n = 3, replicates of analysis) ± SD. a–c for the same analytical parameters, in macro- and microalgae groups, means with different letters are significantly different (*p* ≤ 0.05). A–F for the same analytical parameters, in all algal samples, means with different letters are significantly different (*p* ≤ 0.05); nd - not determined.
